# Differences in Brain Network Topology Based on Alcohol Use History in Adolescents

**DOI:** 10.3390/brainsci13121676

**Published:** 2023-12-05

**Authors:** Haley A. Kirse, Mohsen Bahrami, Robert G. Lyday, Sean L. Simpson, Hope Peterson-Sockwell, Jonathan H. Burdette, Paul J. Laurienti

**Affiliations:** 1 Laboratory for Complex Brain Networks, Wake Forest School of Medicine, Winston-Salem, NC 27101, USA; hkirse@wakehealth.edu (H.A.K.); mbahrami@wakehealth.edu (M.B.); rlyday@wakehealth.edu (R.G.L.); slsimpso@wakehealth.edu (S.L.S.); hpeterso@wakehealth.edu (H.P.-S.); jburdett@wakehealth.edu (J.H.B.); 2 Graduate Program, Wake Forest Graduate School of Arts and Sciences, Integrative Physiology and Pharmacology, Winston-Salem, NC 27101, USA; 3 Department of Radiology, Wake Forest School of Medicine, Winston-Salem, NC 27101, USA; 4 Department of Biostatistics and Data Science, Wake Forest School of Medicine, Winston-Salem, NC 27101, USA

**Keywords:** alcohol, brain networks, adolescents, resting-state connectivity, fMRI

## Abstract

Approximately 6 million youth aged 12 to 20 consume alcohol monthly in the United States. The effect of alcohol consumption in adolescence on behavior and cognition is heavily researched; however, little is known about how alcohol consumption in adolescence may alter brain function, leading to long-term developmental detriments. In order to investigate differences in brain connectivity associated with alcohol use in adolescents, brain networks were constructed using resting-state functional magnetic resonance imaging data collected by the National Consortium on Alcohol and NeuroDevelopment in Adolescence (NCANDA) from 698 youth (12–21 years; 117 hazardous drinkers and 581 no/low drinkers). Analyses assessed differences in brain network topology based on alcohol consumption in eight predefined brain networks, as well as in whole-brain connectivity. Within the central executive network (CEN), basal ganglia network (BGN), and sensorimotor network (SMN), no/low drinkers demonstrated stronger and more frequent connections between highly globally efficient nodes, with fewer and weaker connections between highly clustered nodes. Inverse results were observed within the dorsal attention network (DAN), visual network (VN), and frontotemporal network (FTN), with no/low drinkers demonstrating weaker connections between nodes with high efficiency and increased frequency of clustered nodes compared to hazardous drinkers. Cross-sectional results from this study show clear organizational differences between adolescents with no/low or hazardous alcohol use, suggesting that aberrant connectivity in these brain networks is associated with risky drinking behaviors.

## 1. Introduction

It is well known that the brain undergoes a variety of developmental changes during adolescence and even into early adulthood, leading to both structural and functional changes, along with corresponding changes in behavior and cognition [1,2,3,4,5,6,7,8,9]. During this period of vital neurodevelopment, accumulating research suggests that the adolescent brain is increasingly vulnerable to adverse experiences due to sensitive periods of adolescent brain development that are heavily shaped by experience and environment, leading to deviations in typical development and potentially resulting in long-standing changes in brain structure and function [10,11,12]. Associated with the remodeling of frontal and limbic brain regions, adolescence also marks a period of increased risk taking, including initiating the use of illicit substances such as alcohol [13,14].

Alcohol is the most commonly used illicit substance among youth in the United States, with over 5.9 million adolescents aged 12–20 reporting consuming more than “just a few sips” of alcohol in the past month, according to a 2021 study by the National Institute on Alcohol Abuse and Alcoholism [15]. A large number of studies have linked adolescent alcohol use to impaired cognitive, emotional, and behavioral functioning, including impaired attention and working memory [16], as well as worsened verbal learning and memory, cognitive flexibility, and learning capabilities [17,18,19,20]. Adolescent alcohol use is also associated with poor educational performance, deficits in decision making, and worse cognitive flexibility [21]. Additionally, alcohol consumption in adolescence has been linked to alterations in brain structure and function. Youth who consume alcohol heavily exhibit accelerated decreases in frontal and temporal gray matter, as well as stunted development of white matter in cortical areas [21,22,23,24]. Compared to control, adolescents with an alcohol use disorder had smaller prefrontal cortex and hippocampal gray matter volumes [25,26,27]. During working memory tasks, adolescents with heavy drinking behaviors exhibited decreased activation within the precuneus, precentral gyrus, and occipital areas, as well as increased activation within the parietal lobe [27,28,29]. Adolescent binge drinkers have also been shown to exhibit increased activation within the limbic brain regions during decision-making tasks (the Iowa Gambling task) compared to non-drinking adolescents [27,30]. Other activation studies have found decreases in activation within the frontal, temporal, and parietal cortices during response inhibition tasks [27,31]. These alcohol-associated alterations in cognition, behavior, and brain structure have also been suggested to cause permanent changes in the typical neurodevelopment of the adolescent brain, leading to long-term cognitive deficits and increased vulnerability to developing an alcohol use disorder (AUD) later in life [22,32,33].

Despite numerous studies investigating the effects of alcohol use history (AUH) on behavior, cognition, and brain structure, little is known about how heavy alcohol use in adolescence affects functional brain network organization. Functional network analyses have proven to be a popular tool used to study resting-state functional magnetic resonance imaging (rs-fMRI) [34] by investigating fluctuations in synchronous activity in blood oxygen level-dependent (BOLD) signals [35]. Brain networks provide a way of assessing a variety of behavioral, emotional, and cognitive processes, making them key analytic tools for better understanding typical and non-typical brain function and organization [36]. Analyses of brain networks using rs-fMRI have been performed in a number of studies examining patterns in functional connectivity in a variety of adolescent populations, exploring research questions including how the organization of brain networks changes with age [37,38,39,40], as well as how the brain network organization differs in adolescents with attentional-deficit hyperactivity disorder (ADHD) [41,42,43] and autism spectrum disorder (ASD) [44,45,46].

More recently, research has begun to examine associations between alcohol and other substance use and functional brain organization in adolescents [33,47,48]. Studies analyzing resting-state networks (RSN) built from rs-fMRI in adolescent heavy alcohol users have found weaker functional connectivity in networks involved with emotions, social behaviors, and self-referential thoughts [33] and hyperconnectivity within the sensorimotor network in adolescent female heavy alcohol consumers [48]. Additional studies have found that heavy alcohol use in adolescence is associated with dysfunction in brain areas governing inhibitory control and emotion/stress responses [49,50]. Not all researchers agree with the described findings; given the diverse findings from this limited number of studies [21], more research is needed to investigate associations between alcohol consumption and RSN organization in adolescence. Therefore, the aim of this study was to supplement current knowledge on the association between brain function and alcohol use by comparing RSN organization in adolescents with no-to-low levels of alcohol consumption and adolescents who exhibit hazardous drinking behaviors.

In order to investigate cross-sectional differences in RSN organization in adolescents with no-to-low or hazardous alcohol consumption history, we performed graph theory-based network analyses on baseline rs-fMRI data from participants in the National Consortium on Alcohol and NeuroDevelopment in Adolescence (NCANDA) study. As described above, heavy alcohol use has been linked to a diverse range of behavioral, cognitive, and neurological effects, implicating numerous distinct brain regions. Across the field of alcohol research, most studies have focused on specific brain regions, neglecting global function and interactions between discrete brain areas. This may partially explain the divergent findings in the literature. In the current study, we chose to perform analyses on eight RSNs in an attempt to identify associations between alcohol use and network organization across the brain. Collectively, these eight RSNs covered the entire brain and were constructed by building one static network spanning the whole brain, which was subsequently parcellated into eight functional derived subnetworks/RSNs for more detailed analyses. A major strength of this novel methodology is that it allows for the calculation of network metrics from the full brain, not just within individual RSNs. The brain is a complex system of interacting subsystems; analyzing subnetworks in the context of the whole brain captures interactions between subsystems and can identify potential compensatory mechanisms beyond the analyzed subnetwork. These important components of brain function are overlooked when analyzing subnetworks independently [51,52,53]. We examined drinking group differences in network organization of the default mode network (DMN), central executive network (CEN), salience network (SN), dorsal attention network (DAN), sensorimotor network (SMN), basal ganglia network (BGN), fronto-temporal network (FTN), and the visual network (VN). In examining the topologies of eight RSNs that collectivity covers the entire brain, our goal was to provide more holistic insight into the brain networks that may be vulnerable to heavy alcohol use, capturing both expected and unexpected differences in brain network topology across the brain. For network topological analyses, we examined the global efficiency (GE) and clustering coefficient (CC) of the eight RSNs across all participants, as these network metrics characterize distributed information processing and regional specificity, respectively [14,54,55,56]. Results from this study may provide novel insight into how brain organization differs between adolescents with no/low or hazardous alcohol consumption behaviors, although due to the cross-sectional nature of this analysis, causality cannot be determined in this study. The findings reported could be brain signatures that predispose one to hazardous drinking or could be a consequence of hazardous drinking. Nevertheless, this work will provide neurobiological targets for future studies examining causal relationships.

## 2. Materials and Methods

### 2.1. Participants

NCANDA recruited participants across five data collection sites, including the University of Pittsburgh Medical Center, the University of California at San Diego (UCSD), Duke University Medical Center, SRI International (SRI), and Oregon Health & Science University (OHSU). In total, NCANDA utilized a longitudinal design to recruit 831 participants aged 12–21, with data acquisition occurring at the baseline visit and at the three yearly follow-ups [57]. In this current study, we used rs-fMRI data from 234 NCANDA participants, exactly replicating the participants and data used in a prior study conducted by Muller-Oehring, in which participants were divided into two groups based on drinking levels (no/low or hazardous) and matched for age and sex while also maintaining effect size [33]. Of this sample, 117 met the criteria for no/low drinking, and 117 were categorized as hazardous drinkers based on the National Institute of Alcohol Abuse and Alcoholism (NIAAA) risky drinking guidelines described in Brown et al., 2015 [57]. We used the same matched participant dataset in our analyses to compare our study’s whole-brain network analyses with their work examining seed-based connectivity in this population. The NIAAA risky drinking criteria used in NCANDA were adjusted based on age and sex. For example, adolescents, regardless of sex, ages 12–15 should never have consumed greater than five alcoholic beverages in their lifetime, whereas adolescents aged 18–21 should not exceed 51 lifetime alcoholic drinks. These criteria are further limited based on sex; for instance, boys aged 12–13 cannot surpass three drinks per occasion, and boys older than 20 cannot consume more than five drinks per occasion. NIAAA criteria for no/low drinkers is outlined in Table 1. Participants who exceeded the no/low drinking use criteria were required to meet all other entry criteria but were additionally permitted to exceed nicotine and marijuana exposure criteria [57].For full participant demographics at baseline, see Table 2. We were granted access to this uniquely large dataset by NCANDA and the NIAAA; additionally, the Wake Forest University School of Medicine Institutional Review Board (IRB) approved this study as exempt as all participant data was deidentified. The data were accessed via Amazon Web Services (AWS), with specific details provided in the Supplementary Materials.

### 2.2. MRI Acquisition and Processing

MRI data were acquired across the five NCANDA data collection sites: three sites used 3T GE Discovery MR750 scanners (UCSD, SRI, and Duke University), and two sites used 3T Siemens TIM TRIO scanners (OHSU, University of Pittsburgh). The data collection sites varied slightly on MRI acquisition protocols based on the scanner used at that site, specifically for T1 structural acquisition (see Supplementary Material of [48] for specific MRI acquisition protocol). The rs-fMRI protocol was the same across sites (time of repetition (TR) = 2200 ms, echo time (TE) = 30 ms, 274 volumes, 10 min scan length). For full details on NCANDA MRI acquisition, see (Muller-Oehring et al., 2018). The NCANDA group provided raw participant MRI imaging data. Unless otherwise stated, MRI preprocessing was completed using Statistical Parametric Mapping 12 (SPM 12, Welcome Trust Center, London, UK: www.fil.ion.ucl.ac.uk/spm/, accessed on 14 November 2018). For full details on image processing and brain network generation, please reference the Supplementary Materials.

### 2.3. Statistical Analysis

We used a mixed regression model [51,52,53], as implemented in the WFU_MMNET toolbox [51] for brain networks, to statistically examine if/how hazardous drinking impacts the studied RSNs. The mixed model allowed examining the differences in each RSN topology between the hazardous and no/low drinking groups. Specifically, we first made a binary variable that separated the hazardous and no/low drinking groups as our main covariate of interest (COI). We then used the mixed regression model to examine if/how this covariate affects the relationship between the connectivity and topology of each RSN. The mixed regression model is basically a two-part model that quantifies the relationship between the probability (presence/absence) and strength (correlation values) of brain connections as the outcome (dependent) variables and network and non-network variables as independent variables. The network variables used in this study were the average global efficiency (GE) and clustering coefficient (CC). As described above, the primary non-network covariate of interest (COI) in this study was drinking group membership (no/low drinkers = 0, hazardous drinkers = 1). Additional non-network covariates were included to minimize confounding effects on our results. To control for any side effects that eluded our quantile normalization procedure (see Supplementary Materials), the NCANDA data collection site was included as a covariate (with five levels, coding the five MRI collection sites) in the models. In addition, the spatial Euclidean distance and squared distance between network nodes were included to account for spatial bias in connectivity between nodes in close physical proximity [60]. Network topology analyses were conducted on the whole brain as well as within each RSN. To examine the impacts of our COI on both global (i.e., whole brain) and local (RSNs) networks simultaneously, we used additional binary variables (as independent variables) that separated each RSN from the rest of the brain.

In order to examine local group differences in network topology in individual RSNs, we modeled the interactions between network and non-network variables using two-way (GE/CC × COI) and three-way interactions (GE/CC × COI × RSN) for both connection probability and strength models [51,52,53]. These two-way and three-way interactions were modeled for each of the eight RSNs individually (BGN, CEN, DAN, DMN, SMN, SN, FTN, and VN) in eight separate analyses (where each analysis included running the two-part mixed-effects model, capturing probability and strength, respectively). The application of these regional and whole-brain interaction analyses allowed for both local and global comparisons of brain topology between drinking groups, as group differences in the network topology may not be uniform throughout the brain. The estimates and statistical inferences (i.e., *p*-values) obtained for appropriate two-way interactions determined if the connectivity/topology in the ‘remainder of the brain’ was different between hazardous and no-to-low drinkers. ‘Remainder of the brain’ simply means all brain regions outside of the specific subnetwork being assessed However, as in [61], to identify the difference within the studied RSN in each analysis (e.g., DMN), we performed post hoc analyses on already estimated residuals of the two-way and three-way interactions. Contrast statements were applied to the results from the linear regression analyses to identify significant group differences within individual RSNs. Results from post hoc analyses are presented in the following results section, emphasizing findings comparing within-network topology organizations between no/low and hazardous drinkers. Full tables with results from all interactions are located in the Supplementary Materials. For more details on the interactions and contrast statements produced by this modeling framework, please refer to Bahrami et al., 2019, and Bahrami et al., 2022.

## 3. Results

### 3.1. Mixed-Effects Results from Connection Probability Models

**Figure 1:**
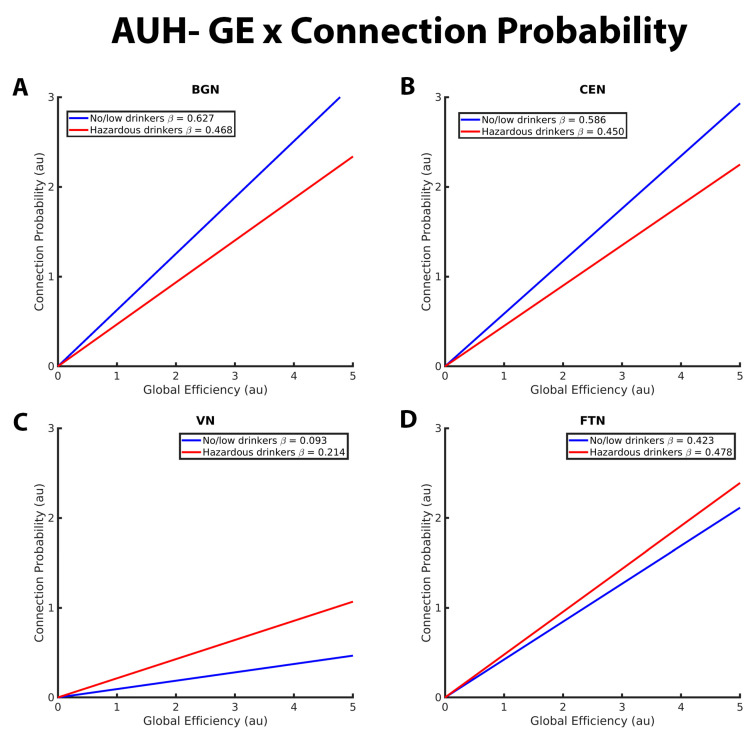
These graphs illustrate the association between global efficiency, connection probability, and alcohol use history (no/low or hazardous) within the (**A**) basal ganglia network (BGN), (**B**) central executive network (CEN), (**C**) visual network (VN), and (**D**) fronto-temporal network (FTN) individually. Alcohol use history (AUH) is characterized as a binary variable. Both drinking groups, no/low drinkers (blue line) and hazardous drinkers (red line) are represented by a ‘best-fit’ slope to most clearly visualize group disparities in the relationship between GE and connection probability within each network. This figure demonstrates a positive global efficiency (GE)-connection probability for both drinking groups within the BGN, CEN, VN, and FTN. This relationship is significantly more positive within the BGN and CEN for no/low drinkers, whereas hazardous drinkers exhibit a stronger GE-connection probability relationship within the VN. There were no significant group AUH group differences in the GE-connection probability relationship within the FTN. The *x* and *y* axes are normalized using arbitrary units. Each Beta β estimate represents the slope of the corresponding line.

**Figure 2:**
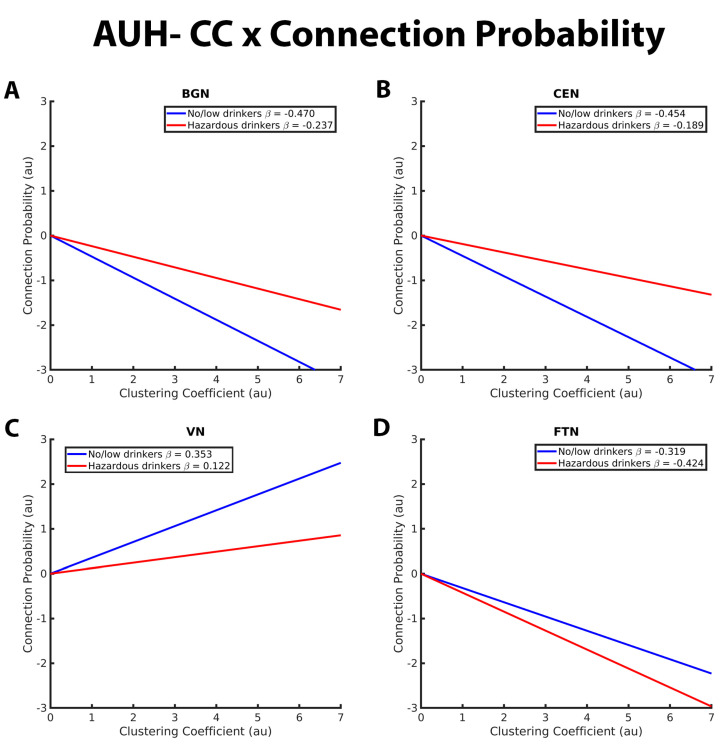
These graphs illustrate the association between clustering coefficient, connection probability, and alcohol use history (no/low or hazardous) within the (**A**) basal ganglia network (BGN), (**B**) central executive network (CEN), (**C**) visual network (VN), and (**D**) fronto-temporal network (FTN) individually. Alcohol use history (AUH) is characterized as a binary variable. Both drinking groups, no/low drinkers (blue line) and hazardous drinkers (red line) are represented by a ‘best-fit’ slope to most clearly visualize group disparities in the relationship between GE and connection probability within each network. The clustering coefficient (CC)-connection probability relationship is negative in both groups in the BGN, CEN, and FTN and positive within the VN. No/low drinkers exhibit a significantly stronger negative relationship between CC and connection probability within the BGN and CEN and a stronger positive relationship between CC and connection probability within the VN. Within the FTN, no/low drinkers demonstrated a weaker negative CC-connection probability relationship compared to hazardous drinkers. The *x* and *y* axes are normalized using arbitrary units. Each Beta β estimate represents the slope of the corresponding line.

**Figure 3:**
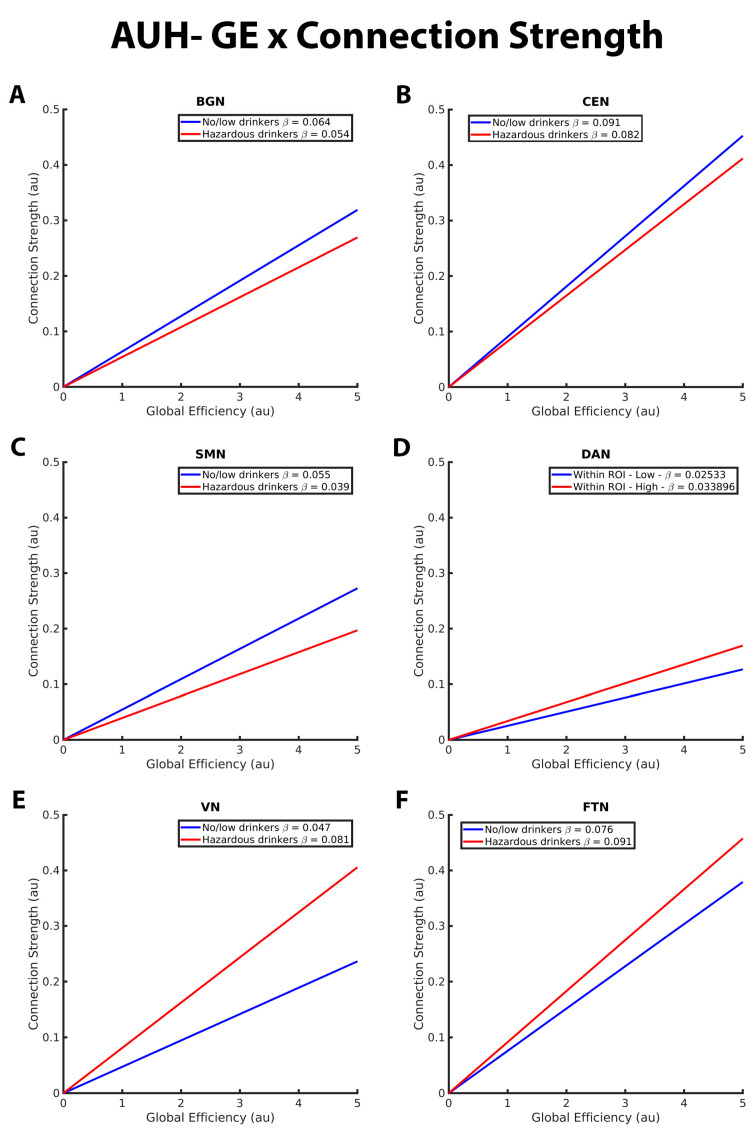
These graphs illustrate the association between global efficiency (GE), connection strength, alcohol use history (no/low or hazardous) within the (**A**) basal ganglia network (BGN), (**B**) central executive network (CEN), (**C**) sensorimotor network (DAN), (**D**) dorsal attention network (DAN), (**E**) visual network (VN), and (**F**) fronto-temporal network (FTN) individually. Alcohol use history (AUH) is characterized as a binary variable. Both drinking groups, no/low drinkers (blue line) and hazardous drinkers (red line) are represented by a ‘best-fit’ slope to most clearly visualize group disparities in the relationship between GE and connection strength within each network. This figure illustrates an overall positive association between GE and connection strength. However, no/low drinkers show a stronger positive GE-connection strength relationship than hazardous drinkers within the BGN, CEN, and SMN but a weaker positive GE-connection strength relationship within the DAN, VN, and FTN. The *x* and *y* axes are normalized using arbitrary units. Each Beta β estimate represents the slope of the corresponding line.

**Figure 4:**
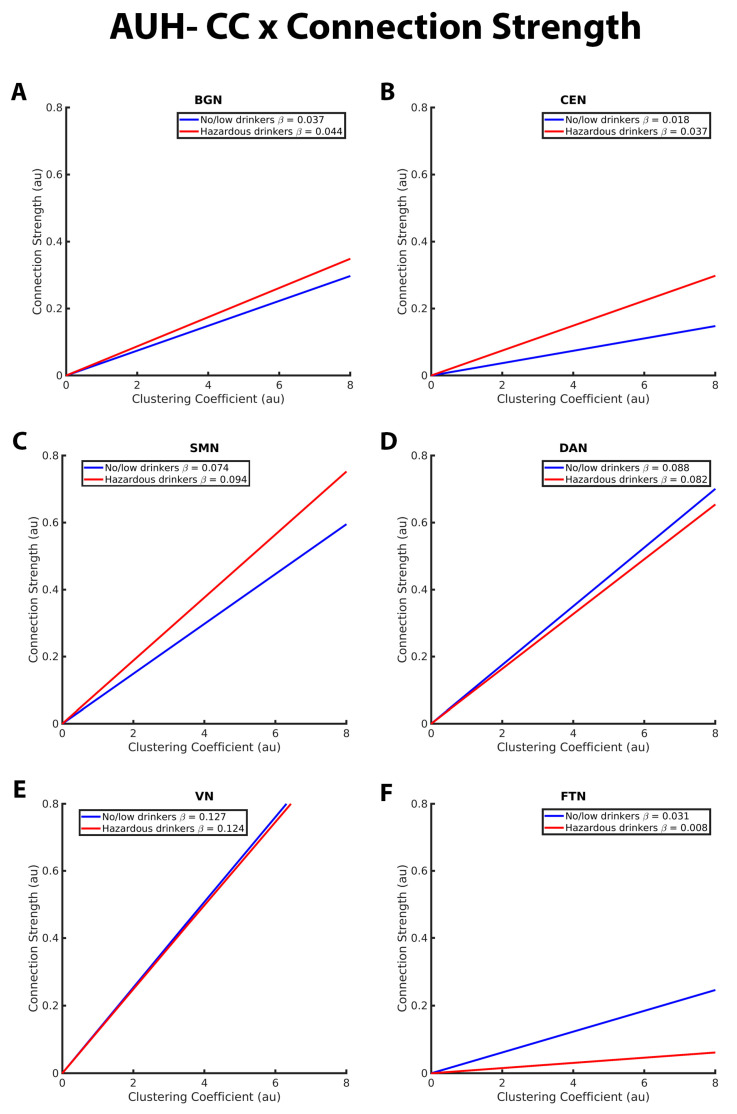
These graphs illustrate the association between clustering coefficient (CC), connection strength, alcohol use history (no/low or hazardous) within the (**A**) basal ganglia network (BGN), (**B**) central executive network (CEN), (**C**) sensorimotor network (DAN), (**D**) dorsal attention network (DAN), (**E**) visual network (VN), and (**F**) fronto-temporal network (FTN) individually. Alcohol use history (AUH) is characterized as a binary variable. Both drinking groups, no/low drinkers (blue line) and hazardous drinkers (red line) are represented by a ‘best-fit’ slope to most clearly visualize group disparities in the relationship between CC and connection strength within each network. This figure illustrates an overall positive association between CC and connection strength. However, no/low drinkers show a stronger positive CC-connection strength relationship than hazardous drinkers within the BGN, CEN, and SMN. Within the FTN, no/low drinkers had a stronger positive CC-connection strength relationship compared to hazardous drinkers. There were no significant group differences in the CC/connection strength relationship within the VN and DAN. The *x* and *y* axes are normalized using arbitrary units. Each Beta β estimate represents the slope of the corresponding line.

Within-network analyses revealed significant group differences between no/low and hazardous in the relationship between GE and connection probability within the BGN, CEN, and VN. Additional significant group differences in the connection probability-CC interaction were observed within the BGN, CEN, VN, and FTN. There were no significant group differences in CC or GE within the SN, SMN, DMN, and DAN (Table 3). Details on these significant group differences in RSN topology are outlined in the following paragraphs. Figure 1, Figure 2, Figure 3 and Figure 4 are an extension of Table 3 and Table 4 by illustrating the directionality and slope of interactions for each drinking group. Although each figure was made using the full interaction results for each RSN analysis, the beta estimates provided in each graph represent the slope for the respective drinking group. Additionally, the estimate provided in the corresponding table denotes the *difference* in slope between groups, with a *p*-value < 0.05 indicating a significant group difference in slope.

Figure 1 illustrates the group differences in the relationships between connection probability and GE within the BGN, CEN, VN, and FTN. Within the BGN and CEN (Figure 1A,B, respectively), no/low drinkers (blue line) showed a significantly stronger relationship between GE and connection probability relative to hazardous drinkers (red line), as indicated by the steeper positive slope. This suggests that within these RSNs, hazardous drinkers are less likely to have connections between nodes with high global efficiency compared to no/low drinkers. The results for the VN (Figure 1C), however, were reversed, with hazardous drinkers demonstrating a stronger positive relationship between GE and connection probability compared to the no/low drinkers. There were no significant group differences observed within the FTN, as illustrated in Figure 1D by the similarly sloped lines.

Figure 2 depicts group differences in the relationship between CC and connection probability within the BGN, CEN, VN, and FTN, respectively. For the BGN and CEN, there was a significantly stronger negative relationship between CC and connection probability in the no/low drinkers versus the hazardous drinkers. This indicates that the no/low group was less likely to have connections between nodes with high CC compared to the hazardous drinkers (Figure 1A,B). No/low drinkers demonstrated a significantly more positive CC-connection probability relationship within the VN and FTN compared to hazardous drinkers (Figure 1C,D). Contrary to the findings within the BGN, CEN, and FTN, both no/low and hazardous drinkers demonstrated a positive relationship between CC and connection probability within the VN.

### 3.2. Mixed-Effects Results from Connection Strength Models

As with the connection probability model, post hoc analyses significant differences in RSN organization between no/low and hazardous drinkers within several RSNs. Within RSN, differences were observed within the BGN, CEN, SMN, DAN, VN, and FTN, indicating significant group differences in the connection strength-GE relationship within these networks. No/low and hazardous drinkers also exhibited significant group differences in the connection strength-CC relationship within the BGN, CEN, SMN, and FTN. There were no significant group differences observed within the DMN or SN (Table 4).

Figure 3 illustrates the group differences in the relationship between GE and connection strength within the BGN, CEN, SMN, DAN, VN, and FTN. Both no/low drinkers and hazardous drinkers had a positive GE-connection strength relationship for all five RSNs. However, within the BGN, CEN, and SMN, the no/low group (blue line) had a significantly stronger positive relationship between GE and connection strength than hazardous drinkers (red line; Figure 3A–C). Corresponding from GE analyses in the connection probability model, these results indicate that hazardous drinkers had weaker functional connections between highly efficient nodes within the BGN, CEN, and SMN. Inverse results were observed within the DAN, VN, and FTN (Figure 3D–F), with hazardous drinkers demonstrating a significantly stronger positive relationship between GE and connection strength than no/low drinkers, signifying that within these RSNs, no/low drinkers instead exhibited weaker functional connections between high GE nodes.

The CC-connection strength relationships for the BGN, CEN, SMN, DAN, VN, and FTN are graphically depicted in Figure 4. As with GE in the connection strength model, both hazardous and no/low drinkers had a positive relationship between CC and connection strength. Within the BGN, CEN, and SMN RSNs, hazardous drinkers demonstrated a significantly stronger positive CC-connection strength relationship compared to no/low drinkers (Figure 4A–C). Results from FTN analyses demonstrated significant group differences in the connection strength CC relationship in which no/low drinkers exhibited a stronger positive association between strength and CC compared to hazardous drinkers (Figure 3F). The slopes of the CC-strength relationship are nearly the same between the no/low or hazardous drinkers within the DAN and VN, consistent with the lack of significant group differences (Figure 4D,E).

For a summary of significant within-network findings from both connection probability and strength models, please see Table 5.

## 4. Discussion

Using the uniquely large rs-fMRI data from adolescents in the NCANDA project, we utilized cross-sectional analyses to examine if/how hazardous drinking is associated with aberrant topology in functional brain networks compared to no/low drinking adolescents. The purpose of examining associations between alcohol consumption and brain network topology is that functional brain connectivity is proving to be a key factor underlying cognitive, emotional, and behavioral dysfunction [38,56,63,64]. Results from this study demonstrated significant differences in RSN organization between no/low drinkers and hazardous drinkers within the CEN, BGN, SMN, DAN, FTN, and VN. No differences were observed in the SN or the DMN. Although causality cannot be determined from this study’s analyses, these results effectively identify significant differences in brain function across the brain that likely either contributed to the onset of hazardous drinking or are a neurological consequence of adolescent hazardous drinking. These findings expand the current limited knowledge base on how hazardous alcohol consumption in adolescence is associated with functional brain organization. The interpretation and implications of the group differences are discussed below for each RSN.

Results from within-network analyses revealed similar differences in group RSN topology within the CEN, BGN, and SMN. In these RSNs, no/low drinkers exhibited a stronger positive association between connection strength and GE compared to hazardous drinkers. No/low drinkers also had a significantly stronger positive GE-connection probability relationship within the CEN and BGN. These results indicate that no/low drinkers are more likely to have edges connecting highly globally efficient nodes within the CEN, BGN, and SMN. GE, which is the inverse of the average shortest path length between two network nodes [2], is a measure of functional integration and how well the brain can efficiently disseminate information across different brain regions and between RSNs. A network with high GE can efficiently distribute information across distinct brain regions, allowing for more economical and diverse information integration and processing [3,4,5]. The no/low drinkers also had a weaker positive relationship between CC and connection strength compared to hazardous drinkers within the CEN, BGN, and SMN. Additionally, within the CEN and BGN, the no/low drinking group displayed a stronger negative CC-connection strength relationship compared to hazardous drinkers. This indicates that no/low drinkers were less likely to have edges connecting highly clustered nodes within the CEN, BGN, and SMN, and existing edges between high CC nodes in the BGN and CEN were much weaker than hazardous drinkers. CC is a measure of the cliquishness of a network in which the neighboring nodes of an individual node are highly interconnected [54]. This network variable quantifies modules of functional connectivity within a network, thus conveying information on the functional segregation and information processing within a network. A network with high CC would consist of clusters of brain areas that are heavily interconnected; communication within a cluster would be high, with limited communication with nodes outside of the clusters. Therefore, information processing in a network with high CC would be more functionally segregated, allowing for more regionally specialized processing and creating more isolated and homogenous information processing than a network with low CC [54,65,66]. Communication in networks with high clustering is more homogenous due to infrequent connections outside of the clusters, therefore reducing novel information input from other brain areas not included in the clusters [65]. In addition, the ability to quantify the strength of edges in a network provides additional information on network organization and information. A strong edge between two nodes indicates a high likelihood of synchronization in those nodes, and synchronization within the brain has been demonstrated to be an important aspect of normal neural processing [67].

Overall results from the CEN, BGN, and SMN analyses showed that no/low drinkers exhibited stronger, more frequent connections between highly efficient nodes, as well as weaker and fewer edges between highly clustered nodes, supporting a topology within the CEN, BGN, and SMN that enables efficient, widespread integration of diverse neural inputs from distinct areas of the brain, both within and between networks. On the other hand, the CEN, BGN, and SMN of hazardous drinkers are less efficient and ‘cliquish,’ with highly clustered nodes communicating with each other in isolated groups and little integration between groups. These results are especially robust within the CEN and BGN, as our analyses revealed significant group differences in CC and GE in both connection probability and strength models.

Within-network analyses from the VN, DAN, and FTN revealed the opposite group differences in RSN topology compared to analyses from the CEN, BGN, and SMN. No/low drinkers exhibited weaker edges connecting nodes with higher metrics of GE in the VN, DAN, and FTN, with overall fewer edges between high GE nodes in the VN and FTN than hazardous drinkers. For CC, no/low drinkers had more frequent edges between highly clustered nodes within the VN and FTN, with stronger edges connecting clusters within the FTN compared to hazardous drinkers. These results illustrate VN and FTN topologies for no/low drinkers that are significantly less efficient and more functionally isolated from the remainder of the brain, while hazardous drinkers displayed stronger connections between highly efficient nodes and reduced levels of clustering in these networks, resulting in more efficient and integrated network organizations. Similarly, but less pronounced, no/low drinkers had weaker edges connecting high GE nodes within the DAN, thus resulting in a less efficient DAN topology when compared to the hazardous drinking group. The implications of these results, along with a discussion of corresponding literature, are discussed for each RSN in the subsequent paragraphs.

The CEN, primarily consisting of the dorsolateral prefrontal cortex and the lateral posterior parietal cortex, has been investigated in numerous studies for its role in a variety of neuro-psychiatric disorders, including alcohol and substance abuse disorders, as well as in non-disordered alcohol use [23,49,68,69,70,71]. Our findings from this study illustrating a more efficient, integrated CEN topology in no/low drinkers are consistent with several other studies that have found altered function of the CEN in various forms of substance use. A prior study found that decreased functional connectivity and integration both within the CEN and between the CEN and the SN were associated with inhibited distress tolerance and increased cocaine use in adults [47]. Segregation of functional connectivity both within the CEN and between the CEN and other brain networks was found to be a key trait of adults with AUD who relapsed 1 month into abstinence, while those who maintained abstinence from alcohol demonstrated stable coupling of the CEN with other distinct networks [68]. Additionally, altered intra- and internetwork CEN connectivity was found in adults after heavy alcohol intake, indicating that the functional connectivity of the CEN differs between sober and intoxicated states [69]. The association between altered CEN connectivity and heavy alcohol use in adolescence is unsurprising, as it is well-known that youth who engage in risky alcohol and substance use have a reduction in lateral frontal lobe gray matter volume and overall decreased activation of the frontal lobe [23,70]. Results from these studies, along with analyses from this study, suggest that dysfunction of CEN connectivity may result in impaired decision-making and an inability to abstain from risky behaviors such as drug and alcohol use.

The BGN and its component structures, which have been largely studied of its role in motor function, habit formation, and reward-based learning, have been implicated in heavy alcohol use across multiple animal and human studies [72,73,74]. Aberrant connectivity between the basal ganglia and other brain regions, namely the orbitofrontal cortex and motor regions, has been associated with the development of ethanol consumption behaviors and dependence in mice [73,74]. A study by Rzepecki-Smith et al. found that the basal ganglia is vulnerable to acute alcohol consumption, causing significant decreases in the functional connectivity and correlated activity of the frontal–temporal–basal ganglia circuit, leading to cognitive and motor impairments [75]. Additionally, acute alcohol intake has been shown to weaken inter-network functional connectivity between the BGN-SMN and BGN-CEN in adults [69]. Along with our findings of a less efficient, functionally segregated BGN organization in hazardous drinkers, these studies suggest that irregular connectivity and isolation of brain areas within the BGN are associated with heavy alcohol use, as well as with the negative side effects of risky drinking including working memory deficits, decreased inhibition, motor impairments, and an increased vulnerability of developing alcohol dependence.

While the SMN is primarily known for its role in motor control and execution, it has also been implicated in inhibitory control and habit regulation [76,77]. Altered connectivity strength in the pre- and post-central gyri (important regions in SMN) has been tied to risky drinking in adolescence [78], and disruptions in the motor network were found to impact executive function and decision making in adults [69]. However, there are studies demonstrating conflicting associations between risky behaviors and alcohol use and SMN connectivity. Consistent with the findings from this study, Silveira and colleagues found that weaker connectivity and segregation of motor regions were associated with deficits in executive function and increased vulnerability to high-risk drinking in late adolescence [78]. On the contrary, hyperconnectivity within the SMN has been observed in stress-predictive networks in risky-drinking adults [49], risky-drinking adolescents [48], and poor inhibitory control in adolescents with ADHD [41]. As it stands, the association between SMN connectivity and alcohol use is still not well understood; it will be important for future research to assess SMN connectivity to help resolve these discrepancies.

The FTN has major nodes in the orbitofrontal cortex (OFC) and amygdala, which are regions of the brain commonly studied for their role in the negative affect and craving aspects of the addiction cycle [79]. The role the amygdala and OFC play in addiction is complex, and much remains to be learned as to how alcohol abuse may affect OFC and amygdala function. Results from this study found that hazardous drinkers displayed and more efficient and integrated FTN; in line with our results, increased resting-state connectivity of the OFC and the nucleus accumbens has previously been found to be positively correlated with alcohol craving in adults [80]. Compared to adults with normal drinking habits, adults with AUD demonstrated increased activity in the left OFC during alcohol-viewing tasks. Similar results have been identified in adults using nicotine, opiates, and cocaine [81]. Increased connectivity within the OFC and amygdala has been linked to risky behaviors in adolescence [14], as well as in obsessive compulsive behaviors in adults and children [82], suggesting that disruption of proper OFC and amygdala function may play a role in compromised decision-making and alcohol abuse. Conversely, task-based imaging studies have found that reduced amygdala-OFC connectivity is associated with alcohol consumption in adolescence [41,83], as well as in adults with alcohol dependence during cognitive tasks [6]. It has been hypothesized that stronger functional connectivity may be a compensatory mechanism in alcohol and substance misuse in order to counteract the negative neurological effects. That is, heavy drug and alcohol use has been shown to decrease efficiency and increase wiring costs of neural functioning, which can be offset via shortening axonal connections within a network to offset higher metabolic costs in other areas of the brain [48,66,71]. This phenomenon may explain the stronger functional connectivity we observed within the FTN for hazardous drinkers in this study.

The VN, which plays a role in visual processes, is not a common focus of neuroimaging addiction studies; nonetheless, a few studies have implicated the VN in substance use, as well as the development of risky behaviors. Increased connectivity of the VN was identified as a prominent feature of stress-predictive networks in high-risk drinkers [49]. Perturbations in connectivity strength within the visual cortex were found to affect top-down executive function and served as a predictive measure of high-risk drinking in adolescence [78]. Additionally, augmented connectivity in the VN was identified in resting-state brain networks after one session of heavy alcohol consumption in adults [69]. Combined with results from this study in which no/low drinkers exhibited weaker connections between nodes with high efficiency, it can be inferred that hazardous drinking in adolescence is associated with dysfunction in VN connectivity. Similar to the FTN, compensatory mechanisms in the brain offsetting the degenerative effects of alcohol may be a possible explanation for the observed stronger functional connectivity for hazardous drinkers.

While also uncommonly researched in addiction studies at rest, the DAN plays a role in attention and goal-directed behavior and has been linked to aberrant behaviors and substance use. Weaker long-range connectivity (i.e., fewer/weaker connections between high GE nodes) of the DAN has been associated with inattention and impulsivity in children with attention-deficiency/hyperactivity disorder (ADHD) [84]. In addition, the increased connectivity of the DAN and VN has been observed in chronic cannabis users, and DAN connectivity was found to be positively correlated with the severity of cannabis use in adults [85]. To date, we did not find any literature connecting DAN topology and alcohol use in the context of rs-fMRI analyses; however, the study by Sami and colleagues implicated aberrant DAN connectivity with cannabis misuse, thus identifying the DAN as a target for future substance and alcohol abuse studies. Nonetheless, results from this study suggest that hyperconnectivity of the DAN is associated with heavy alcohol use in adolescence. As with the VN and FTN, the increase in connectivity of the DAN may be a compensatory mechanism to offset the negative functional effects of alcohol in other areas of the brain.

Surprisingly, we did not observe any significant group differences in network topology within the DMN or SN. Part of the triple network model of psychopathology, the SN and the DMN (along with the CEN) have been implicated in a number of neuro-psychiatric disorders, including substance and alcohol abuse [47,63,86]. Muller-Oehring found that weaker internetwork connectivity in the DMN was associated with hazardous alcohol consumption in adolescence, a finding reflected in a number of additional alcohol use studies [33,49,71]. Additionally, abnormal SN and DMN connectivity have been implicated in cocaine use in adults [63,87,88]. Although we did not observe significant within-network drinking group differences in DMN topology, results from our three-way interactions revealed that the topological differences observed in the DMN were opposite of that observed in the rest of the brain, suggesting that that DMN topology may be associated with alcohol consumption in adolescence. Due to the overall lower level of alcohol consumption, even within the hazardous drinking group [33], it is possible that group differences in DMN and SN organization are not significant due to relatively low levels of alcohol consumption in adolescence.

This study is not without limitations. NCANDA is a cross-sectional cohort study, and the analyses performed here used only baseline data. Therefore, the true cause and effect of hazardous alcohol use in adolescence on RSN organization and function cannot be determined from these analyses. Additionally, the long-term detriments of alcohol use on typical brain function and development cannot be determined from these results. Further analyses on NCANDA rs-fMRI from yearly follow-up visits will provide further insights into determining whether altered connectivity of brain networks underlies pre-existing vulnerability to risky drinking or occurs as a result of alcohol misuse. In addition, our analyses did not control for other substance use or other mental or behavioral disorders, which may contribute to alterations in brain function. Further research is required to understand how these factors may contribute to differences in network organization. It should also be acknowledged that the overall use of alcohol in the hazardous drinking group was relatively low [6]. Furthermore, results from this data can only be generalized to otherwise healthy non- or hazardous adolescent drinkers. It is also important to note that the FTN occupies brain regions that have a higher likelihood of artifact in BOLD imaging data and should be interpreted with caution.

## 5. Conclusions

Alcohol is the most widely used illicit substance among youth; however, knowledge of how heavy alcohol consumption in adolescence affects normal function and neuromaturation of the brain remains limited. The main aim of this current study was to investigate cross-sectional differences in resting-state functional brain network topology between adolescents with either no-to-low or hazardous alcohol use. Results from this study identified distinct organizational differences between hazardous and no/low drinkers within the CEN, BGN, SMN, DAN, VN, and FTN, indicating that heavy alcohol use in adolescence is associated with deviations in normal RSN function. These topological differences in RSN organization may underlie the behavioral and cognitive deficits associated with hazardous drinking, providing insight into the neurological mechanisms of alcohol misuse and identifying important brain regions to target in future alcohol research and neuromodulatory interventions for alcohol use disorder.

## Figures and Tables

**Table 1:** Drinking criteria for no/low drinkers.

Age	Maximum Drinks Per Occasion: Female	Maximum Drinks Per Occasion: Male	Total Days of Drinking in Lifetime
12–13.9	≤3	≤3	≤5
14–15.9	≤3	≤4	≤5
16–16.9	≤3	≤4	≤11
17–17.9	≤3	≤4	≤23
18–19.9	≤3	≤4	≤51
≥20	≤3	≤5	≤51

**Note:** Drinking criteria for adolescent no/low drinkers based on National Institute on Alcohol Abuse and Alcoholism (NIAAA) guidelines for risky drinking [58]. Adolescents who exceed any of these guidelines were categorized as hazardous drinkers in this study. Participants who exceeded these criteria were additionally permitted to exceed the National Survey on Drug Use and Health thresholds for cigarette and marijuana usage only [59]. Use criteria for nicotine, cannabis, and other substances are outlined in Brown et al., 2015.

**Table 2:** Demographics.

		Hazardous Drinkers	No/low Drinkers	Difference between Matched Groups; P=
Total		117	117	
	Girls/Boys	62/55	62/55	
Age	Girls	18.6 ± 2	18.4 ± 1.9	0.39
	Boys	18.7 ± 1.9	18.4 ± 1.7	
GE/Siemens		80/37	72/45	0.27 *
Pubertal Development Scale		3.7 ± 0.4	3.6 ± 0.4	0.28
Alcohol use	# days lifetime	50.6 ± 75.5	3.1 ± 7.2	*<0.001*
	# days past year	23.2 ± 31.8	1.8 ± 4.8	*<0.001*
Nicotine use	# cigarettes lifetime	11.4 ± 45.3	0.7 ± 4.7	*0.012*
	# cigarettes past year	6 ± 28.1	0.3 ± 2.3	*0.03*
Marijuana use	# days lifetime	10.8 ± 17.7	1 ± 3.9	*0.004*
	# days past year	7.5 ± 16	0.6 ± 2.5	*0.015*
Parental education (years)		17.4 ± 2	17 ± 2	0.19

**Note:** Participant demographics and measures from the National Consortium on Alcohol and NeuroDevelopment in Adolescence (NCANDA). Data given as n = subject count; mean ± standard deviation (SD). * = Chi-square test. Significant *p*-vales are marked in italic.

**Table 3:** Post hoc contrast statements for AUH connection probability.

	Estimate	SE	t Value	*p*-Value
GE × AUH within BGN	−0.1597	0.02648	−6.03	**<0.0001**
CC × AUH within BGN	0.2335	0.03098	7.54	**<0.0001**
GE × AUH within CEN	−0.1364	0.03221	−4.24	**<0.0001**
CC × AUH within CEN	0.2653	0.03452	7.69	**<0.0001**
GE × AUH within SMN	0.04766	0.02970	1.60	0.1085
CC × AUH within SMN	−0.05676	0.03014	−1.88	0.0597
GE × AUH within DAN	0.01286	0.03526	0.36	0.7153
CC × AUH within DAN	0.01209	0.03534	0.34	0.7322
GE × AUH within VN	0.1205	0.05416	2.23	**0.0261**
CC × AUH within VN	−0.2312	0.05165	−4.48	**<0.0001**
GE × AUH within FTN	0.05511	0.03888	1.42	0.1564
CC × AUH within FTN	−0.1051	0.04506	−2.33	**0.0196**
GE × AUH within DMN	0.04298	0.02827	1.52	0.1285
CC × AUH within DMN	−0.02400	0.03110	−0.77	0.4403
GE × AUH within SN	−0.02897	0.04502	−0.64	0.5199
CC × AUH within SN	0.06577	0.04454	1.48	0.1398

**Note:** Post hoc findings on within-network topological characteristics within the BGN, CEN, VN, and FTN driven by alcohol use history (AUH). Asterisks represent interactions between variables. Bolded values denote significant effects within the probability model. *p*-values were adjusted using the adaptive false discovery rate procedure [62]. RSNs with at least one significant within-network interaction from the connection probability model are displayed in Figure 1 and Figure 2. Abbreviations: GE, Global efficiency; CC, clustering coefficient; AUH, alcohol use history; SE, standard error. BGN, basal ganglia network; CEN, central executive network; SMN, sensorimotor network; DAN, dorsal attention network; VN, visual network; FTN, fronto-temporal network; DMN, default mode network; SN, salience network.

**Table 4:** Post hoc contrast statements for AUH connection strength.

	Estimate	SE	t Value	*p*-Value
GE × AUH within BGN	−0.00994	0.002110	−4.71	**<0.0001**
CC × AUH within BGN	0.006438	0.002511	2.56	**0.0104**
GE × AUH within CEN	−0.00821	0.002697	−3.04	**0.0023**
CC × AUH within CEN	0.01878	0.002807	6.69	**<0.0001**
GE × AUH within SMN	−0.01514	0.002187	−6.92	**<0.0001**
CC × AUH within SMN	0.01967	0.002180	9.02	**<0.0001**
GE × AUH within DAN	0.008566	0.003145	2.72	**0.0065**
CC × AUH within DAN	−0.00582	0.003017	−1.93	0.0537
GE × AUH within VN	0.03387	0.003597	9.42	**<0.0001**
CC × AUH within VN	−0.00249	0.003226	−0.77	0.4400
GE × AUH within FTN	0.01567	0.003474	4.51	**<0.0001**
CC × AUH within FTN	−0.02309	0.004042	−5.71	**<0.0001**
GE × AUH within DMN	0.003482	0.002007	1.73	0.0828
CC × AUH within DMN	−0.00071	0.002040	−0.35	0.7281
GE × AUH within SN	−0.00136	0.003514	−0.39	0.6998
CC × AUH within SN	0.003587	0.003371	1.06	0.2873

**Note:** Post hoc findings on within-network topological characteristics within the BGN, CEN, SMN, DAN, VN, and FTN driven by alcohol use history (AUH). Asterisks represent interactions between variables. Bolded values denote significant effects within the strength model. *p*-values were adjusted using the adaptive false discovery rate procedure [62]. RSNs with at least one significant within-network interaction from the connection probability model are displayed in Figure 3 and Figure 4. Abbreviations: GE, Global efficiency; CC, clustering coefficient; AUH, alcohol use history; SE, standard error. BGN, basal ganglia network; CEN, central executive network; SMN, sensorimotor network; DAN, dorsal attention network; VN, visual network; FTN, fronto-temporal network; DMN, default mode network; SN, salience network.

**Table 5:** Summary of primary findings.

Connection Probability/Strength		No/Low vs. Hazardous Drinkers
**BGN**	CP- Efficiency relation in BGN	* more positive for no/low than hazardous
	CP- Clustering relation in BGN	* more negative for no/low than hazardous
	CS- Efficiency relation in BGN	* more positive for no/low than hazardous
	CS- Clustering relation in BGN	* more positive for hazardous than no/low
**CEN**	CP- Efficiency relation in CEN	* more positive for no/low than hazardous
	CP- Clustering relation in CEN	* more negative for no/low than hazardous
	CS- Efficiency relation in CEN	* more positive for no/low than hazardous
	CS- Clustering relation in CEN	* more positive for hazardous than no/low
**SMN**	CP- Efficiency relation in SMN	no difference
	CP- Clustering relation in SMN	no difference
	CS- Efficiency relation in SMN	* more positive for no/low than hazardous
	CS- Clustering relation in SMN	* more positive for hazardous than no/low
**DAN**	CP- Efficiency relation in DAN	no difference
	CP- Clustering relation in DAN	no difference
	CS- Efficiency relation in DAN	* more positive for hazardous than no/low
	CS- Clustering relation in DAN	no difference
**VN**	CP- Efficiency relation in VN	* more positive for hazardous than no/low
	CP- Clustering relation in VN	* more positive for no/low than hazardous
	CS- Efficiency relation in VN	* more positive for hazardous than no/low
	CS- Clustering relation in VN	no difference
**FTN**	CP- Efficiency relation in FTN	no difference
	CP- Clustering relation in FTN	* more positive for no/low than hazardous
	CS- Efficiency relation in FTN	* more positive for hazardous than no/low
	CS- Clustering relation inFTN	* more positive for no/low than hazardous

Abbreviations: BGN, basal ganglia network; CEN, central executive network; SMN, sensorimotor network; DAN, dorsal attention network; VN, visual network; FTN, fronto-temporal network; CP, connection probability; CS, connection strength. * denotes a significant interaction.

## Data Availability

The data analyzed in this study are publicly available via application at http://www.ncanda.org, accessed on 8 October 2020.

## Supplementary Materials

The following supporting information can be downloaded at https://www.mdpi.com/article/10.3390/brainsci13121676/s1. Supplemental Table S1: Subnetwork node assignments; Supplemental Figure S1: Quantile normalization procedure; Table S2: Independent variables used for mixed-effects regression analyses; Table S3: Whole-brain regression results for AUH; Table S4: Regression results for AUH connection probability; Table S5 Regression results for AUH connection strength; Table S6: Full mixed-effects results for Basal Ganglia Network (BGN) Connection Probability Model; Table S7: Full mixed-effects results for Basal Ganglia Network (BGN) Connection Strength Model; Table S8: Full mixed-effects results for Central Executive Network (CEN) Connection Probability Model; Table S9: Full mixed-effects results for Central Executive Network (CEN) Connection Strength Model; Table S10: Full mixed-effects results for Dorsal Attention Network (DAN) Connection Probability; Table S11: Full mixed-effects results for Dorsal Attention Network (DAN) Connection Strength Model; Table S12: Full mixed-effects results for Default Mode Network (DMN) Connection Probability Model; Table S13: Full mixed-effects results for Default Mode Network (DMN) Connection Strength Model; Table S14: Full mixed-effects results for Fronto-Temporal Network (FTN) Connection Probability Model; Table S15: Full mixed-effects results for Fronto-Temporal Network (FTN) Connection Strength Model; Table S16: Full mixed-effects results for Sensorimotor Network (SMN) Connection Probability Model; Table S17: Full mixed-effects results for Sensorimotor Network (SMN) Connection Strength Model; Table S18: Full mixed-effects results for Salience Network (SN) Connection Probability Model; Table S19: Full mixed-effects results for Salience Network (SN) Connection Strength Model; Table S20: Full mixed-effects results for Visual Network (VN) Connection Probability Model; Table S21: Full mixed-effects results for Visual Network (VN) Connection Strength Model. Supplemental references [89,90,91,92,93,94,95].
