# Supplementary material for: Differences in Brain Network Topology Based on Alcohol Use History in Adolescents
Source: Brain Sci. 2023 Dec 5;13(12):1676. doi: 10.3390/brainsci13121676 (PMC10741456; doi:10.3390/brainsci13121676)
Supplement: Supplementary file 1 [file brainsci-13-01676-s001.zip › brainsci-2651773-supplementary.pdf]

Methods

Participants

Deidentified participant data was released through NCANDA_RELEASE_BASE_RESTINGSTATE_IMAGES_V03, NCANDA_RELEASE_BASE_STRUCTURAL_IMAGES_V03, NCANDA_PUBLIC_BASE_REDCAP_V02 ,NCANDA_PUBLIC_BASE_RESTINGSTATE_V01.

MRI Processing

Image processing started with segmenting the T1-weighted structural images using standard six tissue priors and warping the images to Montreal Neurological Institute (MNI) standard space ({www.mni.mcgill.ca}). The first 10 volumes of the functional scan were removed and the remaining volumes were corrected for slice-time differences, then realigned to the first volume. Motion and other artifacts were removed from the functional images with the independent component analysis-based method Automatic Removal of Motion Artifacts (ICA-AROMA) (Pruim et al., 2015). The previously defined warp to MNI space was then applied to the aligned and slice-time corrected functional images to perform analyses in standard space. To further remove physiological noise and low frequency drift, the functional data was filtered using a standard band pass filter (0.009-0.08Hz). Whole brain gray matter, white matter, and cerebral spinal fluid mean signals, along with the six degrees of freedom (DOF) motion parameters obtained from realignment were regressed from the functional data. The functional images were then parcellated into functionally defined 268 regions based on the Shen atlas (Shen et al., 2013) and the time series from each voxel within a region were in each region of the atlas. For the network analyses applied here, each region served as a network node. For information on nodes assigned to each specific network, please see Supplemental Table 1. A Pearson's (full) correlation was then calculated between each of the functional time series from pair of nodes within the atlas to create a weighted network to be used in our statistical analyses. It is important to note that, in this study, we chose to focus on positive correlation values (setting all negative correlation values to zero) since defining several network measures, including clustering coefficient, in networks containing negative correlation values is computationally challenging. Additionally, the interpretation of several network variables varies greatly in networks that do or do not contain negative edges (Bahrami et al., 2022; Fraiman et al., 2009; Parente et al., 2018).

While acquiring data at five sites has provided more statistical power and generalizability, our initial assessments demonstrated significant differences in network topology between data collected at the different sites as well as from different scanners. The site effect resulted in different network edge distributions across the five sites (Supplemental Figure S1). Muller-Oehring and colleagues also noted significant differences in seed-based connectivity between scanner types and developed a method to correct this, however this seed-based correction method was not applicable to brain networks. Therefore, we used quantile normalization as an alternative correction method that could be appropriately applied to brain networks to remove site and scanner effects. Quantile normalization was originally developed for removing batch effects in gene expression microarrays (Amaratunga & Cabrera, 2001; Bolstad et al., 2003) and has become a popular technique to remove effects that result in different distributions. Specifically, we used the following steps to apply quantile normalization to our data: i) combining all weighted networks within each site and ranking them; ii) averaging the connectivity values

that occupy the same rank across the five sites; iii) replacing all connectivity values occupying the same rank with the computed average in ii; and iv) Rearranging the updated connectivity values into their original place in each network. Supplemental figure 1 illustrates the distributions for a given brain region's connectivity (correlation values representing weighted edges) (Shen region 77) across the five sites, before and after correcting for site effects using quantile normalization. We also incorporated site as a confounding variable in our mixed-effects regression modeling detailed below.

Statistical Modeling Framework

We used a two-part mixed-effects regression framework (Bahrami et al., 2019a; Simpson & Laurienti, 2015), suited for testing hypotheses on brain networks, to examine if/how heavy alcohol drinking, as our desired covariate, impacts the desired RNs. This multivariate model also allowed for the inclusion of possible confounding covariates including age, sex, and site effects, as well as endogenous network-related confounding effects, such as the spatial distance between the brain regions. This model then allowed quantifying the relationship between the desired and confounding covariates, as independent variables, and the probability (presence/absence – Part I) and strength of present brain connections (Part II), as dependent variables, in two separate models, and providing statistical inference for the quantified relationships. Network metrics (CC and GE) were also included as independent covariates in both models, and incorporating their interactions with the main covariates of interest (i.e., the grouping covariate separating the heavy alcohol drinkers from no/low alcohol drinkers) allowed us to examine if/how the relationship between brain connectivity (probability/strength) and network metrics (characterizing topology) was modified by the grouping covariate (i.e., heavy alcohol drinking). Below we have provided more detail about this methodology.

Let R_{ijk} denote a binary variable which is one if the correlation value between node j and node k of the ith participant's network is positive, and zero otherwise. This variable specifies whether a connection exists between node j and node k of the ith child's network. Also, let Y_{ijk} denote a continuous variable for positive correlation values (present connections) between node j and node k of the ith child's network. Then, we can define the following conditional probabilities:

$$\boldsymbol{P}\left(\boldsymbol{R_{ijk}} = \mathbf{1} | \boldsymbol{\beta_r}; \boldsymbol{b_{ri}}\right) = \boldsymbol{p_{ijk}}(\boldsymbol{\beta_r}; \boldsymbol{b_{ri}}) \qquad \text{(S1)}$$

$$\boldsymbol{P}\left(\boldsymbol{R_{ijk}} = \mathbf{0} | \boldsymbol{\beta_r}; \boldsymbol{b_{ri}}\right) = \mathbf{1} - \boldsymbol{p_{ijk}}(\boldsymbol{\beta_r}; \boldsymbol{b_{ri}}) \qquad \text{(S2)}$$

Where p_{ijk} is the probability of having a connection between node j and node k of the ith subject's network, β_r is the fixed effects (population) parameters vector (note that the original paper which introduces this new extension uses θ for fixed effects parameters. We used β as it is a more familiar notation for fixed effect parameters), and b_{ri} is the random-effects parameter vector modeling correlation (dependence) between repeated network features for subject i. If S_{ijk} denotes a variable specifying present connections between node j and node k of the ith participant's network (i.e., S_{ijk} = $[Y_{ijk} | R_{ijk} = 1]$), we can define the following two-part mixed modeling framework for the probability and strength of brain connections:

$$logit\left(p_{ijk}(\boldsymbol{\beta}_r; \boldsymbol{b}_{ri})\right) = \boldsymbol{T}'_{ijk}\boldsymbol{\beta}_r + \boldsymbol{Z}'_{ijk}\boldsymbol{b}_{ri} \qquad \text{(S3)}$$

$$FZT\left(S_{ijk}(\boldsymbol{\beta}_s;\boldsymbol{b}_{si})\right) = \boldsymbol{T}'_{ijk}\boldsymbol{\beta}_s + \boldsymbol{Z}'_{ijk}\boldsymbol{b}_{si} + \boldsymbol{e}_{ijk} \quad \text{(S4)}$$

Where T_{ijk} and Z_{ijk} are design matrices for the fixed- and random-effects, respectively, e_{ijk} captures the random noise (not captured by random effects) in the connection strength between node j and node k of the ith Participant's network, and β_s and b_{si} are analogous parameters to β_r and b_{ri}, respectively, but for the connection strength. Eq. S3 is a logistic regression model that quantifies the relationship between the connection probability and sets of desired covariates. FZT is the Fisher's Z-transform applied to ensure that normality assumption is met. By including network metrics (such as CC and GE) and regional covariates modeling brain RSNs (DMN, SMN, etc.), this framework allows testing hypotheses about the DMN and whole brain connectivity and network metrics and assessing how covariates of interest affect the connectivity and topology at local and global levels. For more detail, see the referenced paper. We used the WFU_MMNET toolbox (Bahrami et al., 2019b) and in-house Matlab scripts to generate the appropriate datasets for this modeling framework, and used SAS v.9.4 to fit the statistical models. 7 separate analyses were run for the 7 desired networks with each analysis using the two-part model for the probability and strength of brain connections.

The differences in the studied RSNs (DMN, CEN, SMN, etc.) between the two groups and were determined by applying appropriate contrast statements (estimates of linear combinations of appropriate covariates) on already estimated residuals in post-hoc analyses. Specifically, to obtain inference about if/how the relationship between network metrics (CC, GE) and brain connectivity within a RSN (e.g., DMN) is modified by the grouping covariate (i.e., if the relationship is different between heavy alcohol drinkers and no/low alcohol drinkers). The contrast statements are provided in Table SX, but, it is important to note that these statements (i.e., combination of parameters) were not used as additional independent variables, and rather were used to test hypotheses (i.e., obtain inference) on combinations of estimated parameters by using their already estimated residuals. A summary of what each important interaction (i.e., interactions that include network measures and the covariate-of-interest, or AUH) shows, and how contrast statements for comparing the RSNs are obtained via the interaction covariates are in the supplemental material of (Bahrami et al., 2022)**.** To better understand the conducted analyses, we have presented the independent covariates used in the analysis that examined the effects of our covariate of interest (AUH), i.e., grouping covariate separating the two groups, on the DMN in supplemental Table 2.

Results

Mixed-Effects Results from Connection Probability Models

There were significant three-way interactions (GE *RSN*AUH and CC *RSN*AUH) in the connection probability model for the BGN, CEN, VN, FTN, SMN, and DMN. A significant three-way interaction indicates that topology comparisons between the RSN and the remainder of the brain were different between the no/low and hazardous drinkers (Table 3). For both the BGN and the CEN, the direction of the group differences in the network topology were in the same direction, but larger than, the reminder of the brain. For the VN, FTN, SMN, and DMN, the direction of the topological differences between drinking groups was opposite of that observed in the remainder of the brain. This is noted by the

reversal of the sign of the estimates (-/+) when comparing the two-way and three-way interactions for each topological variable.

Mixed-Effects Results from Connection Strength Models

For within RSN analyses, the BGN, CEN, SMN, DAN, VN, and FTN all exhibited significant differences between drinking groups for GE in the connection strength model. In addition, all networks except the VN had significant group differences for CC (Table 5). Results from these three-way interactions indicate that topology comparisons between the RSN and the remainder of the brain were different between the no/low and hazardous.

Supplemental Figures

Supplemental Table S1: Subnetwork node assignments

DMN Node ID	CEN Node ID	DAN Node ID	SMN Node ID	SN Node ID	BGN Node ID	VN Node ID	FTN Node ID
3	7	31	23	15	93	42	1
5	8	43	24	16	94	72	2
6	9	44	25	20	95	75	4
10	11	49	26	21	99	76	18
12	14	66	27	28	101	77	51
13	17	67	32	29	103	79	52
48	19	68	33	34	104	80	57
50	22	69	38	35	105	82	58
53	30	71	39	36	117	87	59
54	47	73	40	37	118	98	60
55	70	74	41	46	120	106	92
56	111	78	45	65	121	205	96
63	112	81	61	91	122	208	97
64	114	102	62	144	123	211	107
83	116	113	84	155	124	212	135
85	131	119	89	168	125	213	136
86	139	177	108	169	126	215	185
88	142	178	109	170	127	216	186
90	143	198	110	181	128	236	189
100	147	200	130	188	129	244	194
115	149	203	158	192	132	248	195
134	150	204	159	220	133		201
137	151	206	160	221	217		202
138	152	207	161	249	228		232
140	154	209	162	250	229		234
141	156	210	163		230		235
145	157	214	165		231		
146	164	238	166		233		
148	184	240	167		237		
153	199	241	171		243		
176	246		172		251		
182	247		173		252		
183	253		174		255		
187	268		175		256		
190			179		257		
193			180		258		
196			191		259		
197			218		260		
219			226		261		
222			245		262		
223			254		263		
224					264		
225					265		
227					266		
239					267		
242							

Table S1: Resting-state network node assignments based on methods outlined in (Laurienti et al., 2023). Shen (Yale) atlas was used to define nodes (Shen et al., 2013). Abbreviations: BGN, basal ganglia network; CEN, central

executive network; DMN, default mode network; DAN, dorsal attention network; FTN, fronto-temporal network; SN, salience network; SMN, sensorimotor network; VN, visual network.

Supplemental Figure S1: Quantile Normalization procedure

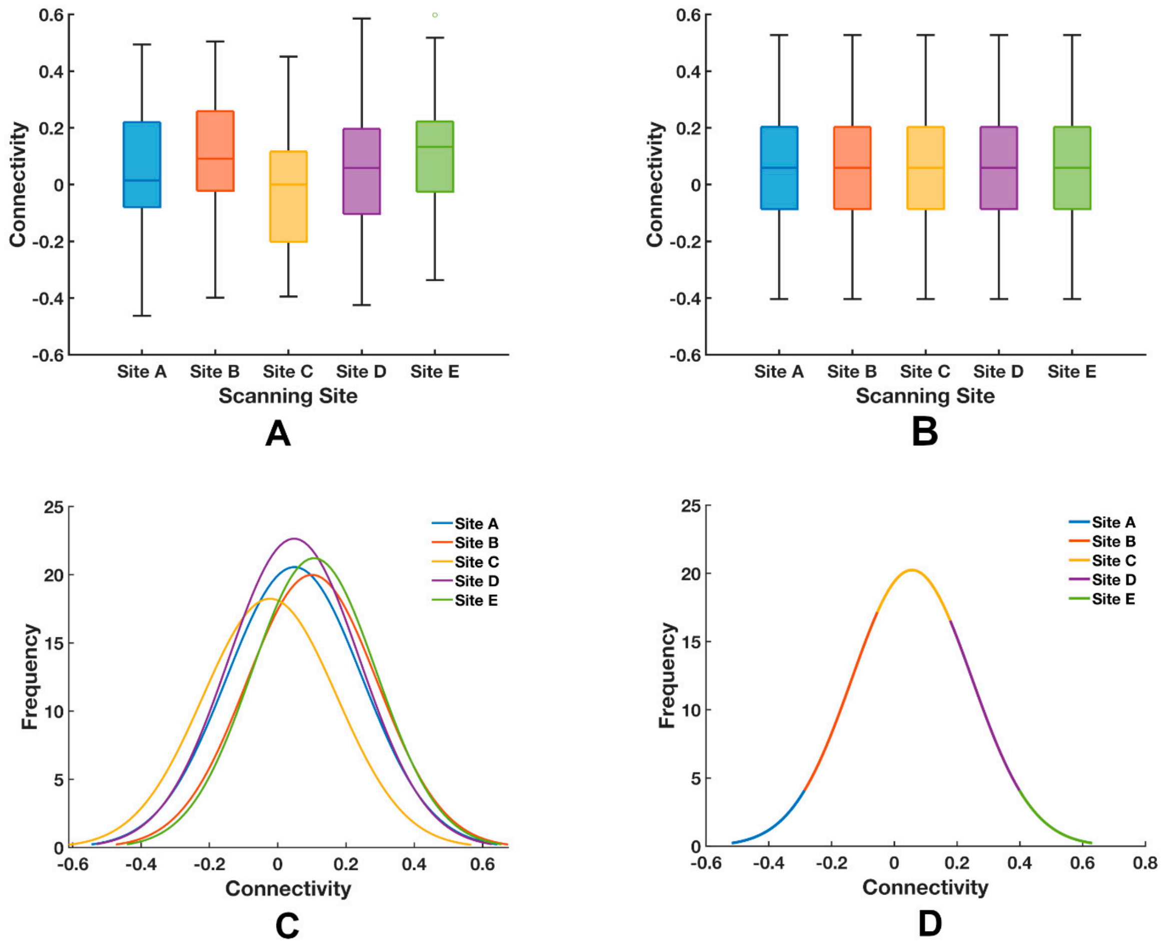

Figure S1: Removing site effects from functional network data of NCANDA. This figure demonstrates the distribution of correlation value of a sample ROI (ROI 77 from Shen Atlas) (Shen et al., 2013) before (A and C) and after (B and D) correcting for site effects using quantile normalization.

Supplemental Table S2: Independent variables used for mixed-effects regression analyses

Covariates	Parameters	
	**Probability Model**	**Strength Model**
**Covariate of Interest**	$\beta_{r,AUH}$	$\beta_{s,AUH}$
**RSN covariate**	$\beta_{r,RSN}$	$\beta_{s,RSN}$
**Network metric covariates**	$\beta_{r,CC}$	$\beta_{s,CC}$
	$\beta_{r,GE}$	$\beta_{s,GE}$
**Interaction covariates**	$\beta_{r,AUH\times RSN}$	$\beta_{s,AUH\times RSN}$
	$\beta_{r,AUH\times CC}$	$\beta_{s,AUH\times CC}$
	$\beta_{r,AUH\times GE}$	$\beta_{s,AUH\times GE}$
	$\beta_{r,RSN\times CC}$	$\beta_{s,RSN\times CC}$
	$\beta_{r,RSN\times GE}$	$\beta_{s,RSN\times GE}$
	$\beta_{r,AUH\times RSN\times CC}$	$\beta_{s,AUH\times RSN\times CC}$
	$\beta_{r,AUH\times RSN\times GE}$	$\beta_{s,AUH\times RSN\times GE}$
**Confounding covariates**	$\beta_{r,Dist}$	$\beta_{s,Dist}$
	$\beta_{r,Dist2}$	$\beta_{s,Dist2}$
	$\beta_{r,Site}$	$\beta_{s,Site}$
**Contrast Statements***	$\beta_{r,AUH\times CC} + \beta_{r,AUH\times RSN\times CC}$	$\beta_{s,AUH\times CC} + \beta_{s,AUH\times RSN\times CC}$
	$\beta_{r,AUH\times GE} + \beta_{r,AUH\times RSN\times GE}$	$\beta_{s,AUH\times GE} + \beta_{s,AUH\times RSN\times GE}$

Table S2: *Contrast statements were not used as additional independent variables. They were rather used in post-hoc analyses to test hypotheses (i.e., obtain inference) on combinations of estimated parameters using their already estimated residuals.

Abbreviations: CC, Clustering Coefficient; GE, Global Efficiency; AUH, Covariate of Interest; RSN, resting state network.

Supplemental Table S3: Whole-Brain regression results for AUH

	Estimate	SE	t Value	P-Value
	**AUH- Connection Probability**			
**GE*AUH**	-0.02608	0.01851	-1.41	0.1589
**CC*AUH**	0.02488	0.02228	1.12	0.2642
	**AUH- Connection Strength**			
**GE*AUH**	0.000498	0.001019	0.49	0.6255
**CC*AUH**	0.001206	0.001424	0.85	0.3971

Table S3: Results are from network analyses from whole-brain regression analyses for alcohol use history (AUH) for both connection strength and probability models. Asterisks represent interactions between variables. There were no significant whole-brain interactions for any of the covariates of interest across either model. P-values were adjusted using the adaptive false discovery rate procedure (Benjamini & Hochberg, 2000).

Abbreviations: GE, Global efficiency; CC, clustering coefficient; AUH, alcohol use history; SE, standard error.

Supplemental Table S4- Regression results for AUH Connection Probability

	Estimate	SE	t Value	P-Value
Basal Ganglia Network (BGN)				
**GE*AUH**	-0.02109	0.01815	-1.16	0.2453
**CC*AUH**	0.01752	0.02166	0.81	0.4187
**GE*AUH*BGN**	-0.1386	0.01973	-7.03	**<.0001**
**CC*AUH*BGN**	0.2160	0.02256	9.57	**<.0001**
Central Executive Network (CEN)				
**GE*AUH**	-0.02511	0.01873	-1.34	0.1802
**CC*AUH**	0.02172	0.02240	0.97	0.3323
**GE*AUH*CEN**	-0.1113	0.02654	-4.19	**<.0001**
**CC*AUH*CEN**	0.2436	0.02663	9.15	**<.0001**
Visual Network (VN)				
**GE*AUH**	-0.02992	0.01942	-1.54	0.1234
**CC*AUH**	0.02984	0.02357	1.27	0.2055
**GE*AUH*VN**	0.1504	0.05072	2.97	**0.0030**
**CC*AUH*VN**	-0.2611	0.04617	-5.65	**<.0001**
Fronto-Temporal Network (FTN)				
**GE*AUH**	-0.02687	0.01839	-1.46	0.1440
**CC*AUH**	0.02617	0.02215	1.18	0.2374
**GE*AUH*FTN**	0.08199	0.03451	2.38	**0.0175**
**CC*AUH*FTN**	-0.1313	0.03949	-3.33	**0.0009**
Sensorimotor Network (SMN)				
**GE*AUH**	-0.02580	0.01811	-1.42	0.1543
**CC*AUH**	0.02294	0.02158	1.06	0.2878
**GE*AUH*SMN**	0.07347	0.02388	3.08	**0.0021**
**CC*AUH*SMN**	-0.07970	0.02145	-3.72	**0.0002**
Default Mode Network (DMN)				
**GE*AUH**	-0.02346	0.01925	-1.22	0.2229
**CC*AUH**	0.02431	0.02364	1.03	0.3037
**GE*AUH*DMN**	0.06644	0.02115	3.14	**0.0017**
**CC*AUH*DMN**	-0.04831	0.02071	-2.33	**0.0196**
Salience Network (SN)				
**GE*AUH**	-0.02777	0.01837	-1.51	0.1306
**CC*AUH**	0.02572	0.02213	1.16	0.2451
**GE*AUH*SN**	-0.00119	0.04130	-0.03	0.9770
**CC*AUH*SN**	0.04005	0.03888	1.03	0.3030
Dorsal Attention Network (DAN)				
**GE*AUH**	-0.02715	0.01885	-1.44	0.1498
**CC*AUH**	0.02626	0.02265	1.16	0.2463

**GE*AUH*DAN**	0.04001	0.03007	1.33	0.1833
**CC*AUH*DAN**	-0.01416	0.02746	-0.52	0.6060

Table S4: Results are from network analyses from all 8 resting-state networks for connection probability. AUH is a binary variable separating no/low and hazardous drinkers. Asterisks represent interactions between variables. Significant effects are bolded. P-values were adjusted using the adaptive false discovery rate procedure (Benjamini & Hochberg, 2000). Abbreviations: GE, Global efficiency; CC, clustering coefficient; AUH, alcohol use history; SE, standard error.

Supplemental Table S5- Regression results for AUH Connection Strength

	Estimate	SE	t Value	P-Value
Basal Ganglia Network (BGN)				
**GE*AUH**	0.001081	0.001052	1.03	0.3040
**CC*AUH**	0.000708	0.001478	0.48	0.6319
**GE*AUH*BGN**	-0.01102	0.001897	-5.81	**<.0001**
**CC*AUH*BGN**	0.005730	0.002092	2.74	**0.0062**
Central Executive Network (CEN)				
**GE*AUH**	0.000684	0.001050	0.65	0.5151
**CC*AUH**	0.000676	0.001487	0.45	0.6496
**GE*AUH*CEN**	-0.00889	0.002535	-3.51	**0.0005**
**CC*AUH*CEN**	0.01810	0.002437	7.43	**<.0001**
Sensorimotor Network (SMN)				
**GE*AUH**	0.001498	0.001131	1.33	0.1850
**CC*AUH**	-0.00025	0.001599	-0.15	0.8776
**GE*AUH*SMN**	-0.01663	0.001929	-8.62	**<.0001**
**CC*AUH*SMN**	0.01991	0.001550	12.85	**<.0001**
Dorsal Attention Network (DAN)				
**GE*AUH**	0.000106	0.001001	0.11	0.9159
**CC*AUH**	0.001683	0.001420	1.19	0.2359
**GE*AUH*DAN**	0.008460	0.003021	2.80	**0.0051**
**CC*AUH*DAN**	-0.00750	0.002712	-2.77	**0.0056**
Visual Network (VN)				
**GE*AUH**	-0.00104	0.000961	-1.08	0.2784
**CC*AUH**	0.002881	0.001370	2.10	**0.0355**
**GE*AUH*VN**	0.03491	0.003501	9.97	**<.0001**
**CC*AUH*VN**	-0.00537	0.002965	-1.81	0.0700
Fronto-Temporal Network (FTN)				
**GE*AUH**	0.000445	0.001019	0.44	0.6620
**CC*AUH**	0.001431	0.001422	1.01	0.3142
**GE*AUH*FTN**	0.01523	0.003358	4.53	**<.0001**
**CC*AUH*FTN**	-0.02452	0.003820	-6.42	**<.0001**
Default Mode Network (DMN)				

**GE*AUH**	0.000914	0.001104	0.83	0.4077
**CC*AUH**	0.000895	0.001415	0.63	0.5271
**GE*AUH*DMN**	0.002568	0.001748	1.47	0.1418
**CC*AUH*DMN**	-0.00160	0.001552	-1.03	0.3014
Salience Network (SN)				
**GE*AUH**	0.000239	0.001031	0.23	0.8163
**CC*AUH**	0.001310	0.001416	0.92	0.3550
**GE*AUH*SN**	-0.00159	0.003394	-0.47	0.6385
**CC*AUH*SN**	0.002278	0.003099	0.73	0.4624

Table S5. Results are from connection strength analyses for all 8 networks. AUH is a binary variable separating no/low and hazardous drinkers. Asterisks represent interactions between variables. Significant effects are bolded. P-values were adjusted using the adaptive false discovery rate procedure (Benjamini & Hochberg, 2000). Abbreviations: GE, Global efficiency; CC, clustering coefficient; AUH, alcohol use history; SE, standard error.

Supplemental Table S6- Full mixed-effects results for Basal Ganglia Network (BGN) Connection Probability Model

Effect	Estimate	SE	t Value	P-Value
**Intercept**	-0.2688	0.01369	-19.64	<.0001
**AUH**	0.006857	0.01344	0.51	0.6100
**BGN**	0.6333	0.008708	72.72	<.0001
**GE**	0.1847	0.01283	14.39	<.0001
**CC**	-0.2299	0.01531	-15.02	<.0001
**Degree**	-0.08587	0.005433	-15.81	<.0001
**Modularity**	0.03379	0.006632	5.10	<.0001
**GE*AUH**	-0.02109	0.01815	-1.16	0.2453
**CC*AUH**	0.01752	0.02166	0.81	0.4187
**AUH*BGN**	0.03694	0.01258	2.94	0.0033
**GE*BGN**	0.4427	0.01388	31.90	<.0001
**CC*BGN**	-0.2404	0.01585	-15.17	<.0001
**GE*AUH*BGN**	-0.1386	0.01973	-7.03	<.0001
**CC*AUH*BGN**	0.2160	0.02256	9.57	<.0001
**Dist**	-0.2090	0.003836	-54.47	<.0001
**Dist2**	0.1799	0.002232	80.60	<.0001
**Site A**	0.008627	0.01948	0.44	0.6578
**Site B**	0.008850	0.01941	0.46	0.6484
**Site C**	-0.01309	0.02018	-0.65	0.5165
**Site D**	-0.00330	0.02085	-0.16	0.8742
**Site E**	0	.	.	.

Table S6: Alcohol use history (AUH): A binary variable representing alcohol use history group (0- no/low, 1- hazardous). Adjusted using the adaptive FDR procedure described in (Benjamini & Hochberg, 2000).

Abbreviations: AUH, alcohol use history; GE, global efficiency; CC, clustering coefficient; Dist, distance; SE, standard error; BGN, basal ganglia network

Supplemental Table S7- Full mixed-effects results for Basal Ganglia Network (BGN) Connection Strength Model

Effect	Estimate	SE	t Value	P-Value
**Intercept**	0.1895	0.003104	61.06	<.0001
**AUH**	0.004943	0.003050	1.62	0.1050
**BGN**	0.03088	0.000762	40.52	<.0001
**GE**	0.02689	0.000743	36.20	<.0001
**CC**	0.07067	0.001044	67.68	<.0001
**Degree**	-0.03825	0.000421	-90.97	<.0001
**Modularity**	-0.01209	0.001506	-8.02	<.0001
**GE*AUH**	0.001081	0.001052	1.03	0.3040
**CC*AUH**	0.000708	0.001478	0.48	0.6319
**AUH*BGN**	-0.00570	0.001100	-5.18	<.0001
**GE*BGN**	0.03689	0.001331	27.72	<.0001
**CC*BGN**	-0.03353	0.001472	-22.79	<.0001
**GE*AUH*BGN**	-0.01102	0.001897	-5.81	<.0001
**CC*AUH*BGN**	0.005730	0.002092	2.74	0.0062
**Dist**	-0.03776	0.000411	-91.83	<.0001
**Dist2**	0.02783	0.000223	125.07	<.0001
**Site A**	0.007507	0.004418	1.70	0.0893
**Site B**	-0.00689	0.004402	-1.56	0.1178
**Site C**	0.000941	0.004579	0.21	0.8371
**Site D**	0.01018	0.004729	2.15	0.0313
**Site E**	0	.	.	.

Table S7: Alcohol use history (AUH): A binary variable representing alcohol use history group (0- no/low, 1-hazardous). Adjusted using the adaptive FDR procedure described in (Benjamini & Hochberg, 2000).

Abbreviations: AUH, alcohol use history; GE, global efficiency; CC, clustering coefficient; Dist, distance; SE, standard error; BGN, basal ganglia network.

Supplemental Table S8- Full mixed-effects results for Central Executive Network (CEN) Connection Probability Model

Effect	Estimate	SE	t Value	P-Value
**Intercept**	-0.2671	0.01373	-19.45	<.0001
**AUH**	0.006756	0.01348	0.50	0.6163
**CEN**	0.7243	0.009239	78.40	<.0001
**GE**	0.1691	0.01324	12.77	<.0001
**CC**	-0.2195	0.01584	-13.86	<.0001
**Degree**	-0.08401	0.005423	-15.49	<.0001
**Modularity**	0.03342	0.006652	5.02	<.0001
**GE*AUH**	-0.02511	0.01873	-1.34	0.1802
**CC*AUH**	0.02172	0.02240	0.97	0.3323
**AUH*CEN**	0.1309	0.01301	10.05	<.0001
**GE*CEN**	0.4172	0.01917	21.77	<.0001
**CC*CEN**	-0.2346	0.01927	-12.18	<.0001
**GE*AUH*CEN**	-0.1113	0.02654	-4.19	<.0001
**CC*AUH*CEN**	0.2436	0.02663	9.15	<.0001
**Dist**	-0.2241	0.003955	-56.65	<.0001
**Dist2**	0.1781	0.002267	78.56	<.0001
**Site A**	0.007729	0.01954	0.40	0.6924
**Site B**	0.008256	0.01947	0.42	0.6715
**Site C**	-0.01322	0.02025	-0.65	0.5139
**Site D**	-0.00441	0.02091	-0.21	0.8331
**Site E**	0	.	.	.

Table S8: Alcohol use history (AUH): A binary variable representing alcohol use history group (0- no/low, 1- hazardous). Adjusted using the adaptive FDR procedure described in (Benjamini & Hochberg, 2000).

Abbreviations: AUH, alcohol use history; GE, global efficiency; CC, clustering coefficient; Dist, distance; SE, standard error; CEN, central executive network.

Supplemental Table S9- Full mixed-effects results for Central Executive Network (CEN) Connection Strength Model

Effect	Estimate	SE	t Value	P-Value
**Intercept**	0.1892	0.003066	61.71	<.0001
**AUH**	0.004858	0.003013	1.61	0.1069
**CEN**	0.06372	0.000904	70.46	<.0001
**GE**	0.02447	0.000742	32.98	<.0001
**CC**	0.07260	0.001050	69.12	<.0001
**Degree**	-0.03798	0.000419	-90.65	<.0001
**Modularity**	-0.01224	0.001488	-8.23	<.0001
**GE*AUH**	0.000684	0.001050	0.65	0.5151
**CC*AUH**	0.000676	0.001487	0.45	0.6496
**AUH*CEN**	0.004251	0.001243	3.42	0.0006
**GE*CEN**	0.06609	0.001851	35.71	<.0001
**CC*CEN**	-0.05414	0.001801	-30.06	<.0001
**GE*AUH*CEN**	-0.00889	0.002535	-3.51	0.0005
**CC*AUH*CEN**	0.01810	0.002437	7.43	<.0001
**Dist**	-0.03906	0.000419	-93.20	<.0001
**Dist2**	0.02722	0.000224	121.67	<.0001
**Site A**	0.007502	0.004365	1.72	0.0856
**Site B**	-0.00689	0.004349	-1.58	0.1130
**Site C**	0.000959	0.004524	0.21	0.8321
**Site D**	0.01032	0.004672	2.21	0.0272
**Site E**	0	.	.	.

Table S9: Alcohol use history (AUH): A binary variable representing alcohol use history group (0- no/low, 1- hazardous). Adjusted using the adaptive FDR procedure described in (Benjamini & Hochberg, 2000).

Abbreviations: AUH, alcohol use history; GE, global efficiency; CC, clustering coefficient; Dist, distance; SE, standard error; CEN, central executive network.

Supplemental Table S10- Full mixed-effects results for Dorsal Attention Network (DAN) Connection Probability

Effect	Estimate	SE	t Value	P-Value
**Intercept**	-0.2673	0.01383	-19.33	<.0001
**AUH**	0.006955	0.01359	0.51	0.6087
**DAN**	0.5025	0.009811	51.21	<.0001
**GE**	0.1803	0.01333	13.53	<.0001
**CC**	-0.2322	0.01601	-14.50	<.0001
**Degree**	-0.08474	0.005413	-15.66	<.0001
**Modularity**	0.03459	0.006703	5.16	<.0001
**GE*AUH**	-0.02715	0.01885	-1.44	0.1498
**CC*AUH**	0.02626	0.02265	1.16	0.2463
**AUH*DAN**	0.01630	0.01385	1.18	0.2394
**GE*DAN**	0.03004	0.02120	1.42	0.1565
**CC*DAN**	0.09423	0.01860	5.07	<.0001
**GE*AUH*DAN**	0.04001	0.03007	1.33	0.1833
**CC*AUH*DAN**	-0.01416	0.02746	-0.52	0.6060
**Dist**	-0.2169	0.003934	-55.12	<.0001
**Dist2**	0.1841	0.002262	81.42	<.0001
**Site A**	0.007359	0.01969	0.37	0.7085
**Site B**	0.008817	0.01962	0.45	0.6531
**Site C**	-0.01339	0.02040	-0.66	0.5115
**Site D**	-0.00513	0.02107	-0.24	0.8077
**Site E**	0	.	.	.

Table S10: Alcohol use history (AUH): A binary variable representing alcohol use history group (0- no/low, 1- hazardous). Adjusted using the adaptive FDR procedure described in (Benjamini & Hochberg, 2000).

Abbreviations: AUH, alcohol use history; GE, global efficiency; CC, clustering coefficient; Dist, distance; SE, standard error; DAN, dorsal attention network.

Supplemental Table S11- Full mixed-effects results for Dorsal Attention Network (DAN) Connection Strength Model

Effect	Estimate	SE	t Value	P-Value
**Intercept**	0.1883	0.003060	61.54	<.0001
**AUH**	0.004732	0.003007	1.57	0.1156
**DAN**	0.09934	0.000991	100.20	<.0001
**GE**	0.02673	0.000707	37.79	<.0001
**CC**	0.06988	0.001003	69.68	<.0001
**Degree**	-0.03788	0.000419	-90.35	<.0001
**Modularity**	-0.01203	0.001485	-8.10	<.0001
**GE*AUH**	0.000106	0.001001	0.11	0.9159
**CC*AUH**	0.001683	0.001420	1.19	0.2359
**AUH*DAN**	0.003808	0.001393	2.73	0.0063
**GE*DAN**	-0.00140	0.002127	-0.66	0.5103
**CC*DAN**	0.01770	0.001822	9.72	<.0001
**GE*AUH*DAN**	0.008460	0.003021	2.80	0.0051
**CC*AUH*DAN**	-0.00750	0.002712	-2.77	0.0056
**Dist**	-0.03792	0.000417	-90.98	<.0001
**Dist2**	0.02820	0.000225	125.50	<.0001
**Site A**	0.007425	0.004356	1.70	0.0883
**Site B**	-0.00688	0.004341	-1.58	0.1130
**Site C**	0.000894	0.004515	0.20	0.8430
**Site D**	0.01012	0.004662	2.17	0.0300
**Site E**	0	.	.	.

Table S11: Alcohol use history (AUH): A binary variable representing alcohol use history group (0- no/low, 1-hazardous). Adjusted using the adaptive FDR procedure described in (Benjamini & Hochberg, 2000).

Abbreviations: AUH, alcohol use history; GE, global efficiency; CC, clustering coefficient; Dist, distance; SE, standard error; DAN, dorsal attention network.

Supplemental Table S12- Full mixed-effects results for Default Mode Network (DMN) Connection Probability Model

Effect	Estimate	SE	t Value	P-Value
**Intercept**	-0.2924	0.01437	-20.35	<.0001
**AUH**	0.009540	0.01411	0.68	0.4991
**DMN**	1.0773	0.007547	142.74	<.0001
**GE**	0.1689	0.01361	12.41	<.0001
**CC**	-0.2567	0.01671	-15.36	<.0001
**Degree**	-0.08206	0.005424	-15.13	<.0001
**Modularity**	0.03903	0.006961	5.61	<.0001
**GE*AUH**	-0.02346	0.01925	-1.22	0.2229
**CC*AUH**	0.02431	0.02364	1.03	0.3037
**AUH*DMN**	-0.02083	0.01041	-2.00	0.0454
**GE*DMN**	0.1289	0.01520	8.48	<.0001
**CC*DMN**	0.4892	0.01508	32.44	<.0001
**GE*AUH*DMN**	0.06644	0.02115	3.14	0.0017
**CC*AUH*DMN**	-0.04831	0.02071	-2.33	0.0196
**Dist**	-0.2234	0.004008	-55.74	<.0001
**Dist2**	0.1809	0.002298	78.70	<.0001
**Site A**	0.004343	0.02045	0.21	0.8318
**Site B**	0.01281	0.02037	0.63	0.5294
**Site C**	-0.01483	0.02119	-0.70	0.4840
**Site D**	-0.01069	0.02188	-0.49	0.6253
**Site E**	0	.	.	.

Table S12: Alcohol use history (AUH): A binary variable representing alcohol use history group (0- no/low, 1- hazardous). Adjusted using the adaptive FDR procedure described in (Benjamini & Hochberg, 2000).

Abbreviations: AUH, alcohol use history; GE, global efficiency; CC, clustering coefficient; Dist, distance; SE, standard error; DMN, default mode network.

Supplemental Table S13- Full mixed-effects results for Default Mode Network (DMN) Connection Strength Model

Effect	Estimate	SE	t Value	P-Value
**Intercept**	0.1870	0.002930	63.84	<.0001
**AUH**	0.004909	0.002879	1.71	0.0881
**DMN**	0.05498	0.000698	78.80	<.0001
**GE**	0.02404	0.000780	30.81	<.0001
**CC**	0.06762	0.000999	67.66	<.0001
**Degree**	-0.03757	0.000402	-93.45	<.0001
**Modularity**	-0.01145	0.001422	-8.05	<.0001
**GE*AUH**	0.000914	0.001104	0.83	0.4077
**CC*AUH**	0.000895	0.001415	0.63	0.5271
**AUH*DMN**	0.005724	0.000963	5.94	<.0001
**GE*DMN**	0.05537	0.001242	44.60	<.0001
**CC*DMN**	-0.00738	0.001117	-6.61	<.0001
**GE*AUH*DMN**	0.002568	0.001748	1.47	0.1418
**CC*AUH*DMN**	-0.00160	0.001552	-1.03	0.3014
**Dist**	-0.03877	0.000423	-91.61	<.0001
**Dist2**	0.02738	0.000223	123.01	<.0001
**Site A**	0.006904	0.004170	1.66	0.0979
**Site B**	-0.00653	0.004156	-1.57	0.1162
**Site C**	0.000812	0.004323	0.19	0.8509
**Site D**	0.009419	0.004464	2.11	0.0349
**Site E**	0	.	.	.

Table S13: Alcohol use history (AUH): A binary variable representing alcohol use history group (0- no/low, 1-hazardous). Adjusted using the adaptive FDR procedure described in (Benjamini & Hochberg, 2000).

Abbreviations: AUH, alcohol use history; GE, global efficiency; CC, clustering coefficient; Dist, distance; SE, standard error; DMN, default mode network.

Supplemental Table S14- Full mixed-effects results for Fronto-Temporal Network (FTN) Connection Probability Model

Effect	Estimate	SE	t Value	P-Value
**Intercept**	-0.2642	0.01380	-19.15	<.0001
**AUH**	0.007832	0.01355	0.58	0.5633
**FTN**	0.6089	0.01554	39.19	<.0001
**GE**	0.1880	0.01300	14.45	<.0001
**CC**	-0.2353	0.01565	-15.03	<.0001
**Degree**	-0.08526	0.005411	-15.76	<.0001
**Modularity**	0.03394	0.006686	5.08	<.0001
**GE*AUH**	-0.02687	0.01839	-1.46	0.1440
**CC*AUH**	0.02617	0.02215	1.18	0.2374
**AUH*FTN**	-0.02428	0.02323	-1.05	0.2959
**GE*FTN**	0.2349	0.02407	9.76	<.0001
**CC*FTN**	-0.08330	0.02676	-3.11	0.0019
**GE*AUH*FTN**	0.08199	0.03451	2.38	0.0175
**CC*AUH*FTN**	-0.1313	0.03949	-3.33	0.0009
**Dist**	-0.2167	0.003916	-55.33	<.0001
**Dist2**	0.1828	0.002260	80.88	<.0001
**Site A**	0.007923	0.01964	0.40	0.6866
**Site B**	0.007733	0.01957	0.40	0.6927
**Site C**	-0.01318	0.02035	-0.65	0.5170
**Site D**	-0.00441	0.02102	-0.21	0.8338
**Site E**	0	.	.	.

Table S14: Alcohol use history (AUH): A binary variable representing alcohol use history group (0- no/low, 1-hazardous). Adjusted using the adaptive FDR procedure described in (Benjamini & Hochberg, 2000).

Abbreviations: AUH, alcohol use history; GE, global efficiency; CC, clustering coefficient; Dist, distance; SE, standard error; FTN, fronto-temporal network.

Supplemental Table S15- Full mixed-effects results for Fronto-Temporal Network (FTN) Connection Strength Model

Effect	Estimate	SE	t Value	P-Value
**Intercept**	0.1897	0.003095	61.29	<.0001
**AUH**	0.004880	0.003041	1.60	0.1086
**FTN**	0.05417	0.001430	37.89	<.0001
**GE**	0.02752	0.000719	38.25	<.0001
**CC**	0.06986	0.001004	69.56	<.0001
**Degree**	-0.03820	0.000418	-91.39	<.0001
**Modularity**	-0.01209	0.001502	-8.05	<.0001
**GE*AUH**	0.000445	0.001019	0.44	0.6620
**CC*AUH**	0.001431	0.001422	1.01	0.3142
**AUH*FTN**	0.002481	0.002125	1.17	0.2431
**GE*FTN**	0.04830	0.002326	20.77	<.0001
**CC*FTN**	-0.03909	0.002527	-15.47	<.0001
**GE*AUH*FTN**	0.01523	0.003358	4.53	<.0001
**CC*AUH*FTN**	-0.02452	0.003820	-6.42	<.0001
**Dist**	-0.03817	0.000412	-92.55	<.0001
**Dist2**	0.02797	0.000224	124.99	<.0001
**Site A**	0.007492	0.004405	1.70	0.0890
**Site B**	-0.00699	0.004390	-1.59	0.1113
**Site C**	0.000947	0.004566	0.21	0.8357
**Site D**	0.01019	0.004715	2.16	0.0306
**Site E**	0	.	.	.

Table S15: Alcohol use history (AUH): A binary variable representing alcohol use history group (0- no/low, 1-hazardous). Adjusted using the adaptive FDR procedure described in (Benjamini & Hochberg, 2000).

Abbreviations: AUH, alcohol use history; GE, global efficiency; CC, clustering coefficient; Dist, distance; SE, standard error; FTN, fronto-temporal network.

Supplemental Table S16- Full mixed-effects results for Sensorimotor Network (SMN) Connection Probability Model

Effect	Estimate	SE	t Value	P-Value
**Intercept**	-0.2774	0.01408	-19.70	<.0001
**AUH**	0.003054	0.01383	0.22	0.8252
**SMN**	0.9485	0.008174	116.04	<.0001
**GE**	0.1792	0.01281	13.99	<.0001
**CC**	-0.2522	0.01525	-16.53	<.0001
**Degree**	-0.08141	0.005420	-15.02	<.0001
**Modularity**	0.03874	0.006823	5.68	<.0001
**GE*AUH**	-0.02580	0.01811	-1.42	0.1543
**CC*AUH**	0.02294	0.02158	1.06	0.2878
**AUH*SMN**	0.09365	0.01148	8.15	<.0001
**GE*SMN**	0.1314	0.01679	7.83	<.0001
**CC*SMN**	0.4250	0.01486	28.60	<.0001
**GE*AUH*SMN**	0.07347	0.02388	3.08	0.0021
**CC*AUH*SMN**	-0.07970	0.02145	-3.72	0.0002
**Dist**	-0.2098	0.003995	-52.51	<.0001
**Dist2**	0.1817	0.002257	80.50	<.0001
**Site A**	0.005290	0.02004	0.26	0.7918
**Site B**	0.009716	0.01997	0.49	0.6265
**Site C**	-0.01507	0.02077	-0.73	0.4679
**Site D**	-0.01091	0.02145	-0.51	0.6108
**Site E**	0	.	.	.

Table S16: Alcohol use history (AUH): A binary variable representing alcohol use history group (0- no/low, 1- hazardous). Adjusted using the adaptive FDR procedure described in (Benjamini & Hochberg, 2000).

Abbreviations: AUH, alcohol use history; GE, global efficiency; CC, clustering coefficient; Dist, distance; SE, standard error; SMN, sensorimotor network.

Supplemental Table S17- Full mixed-effects results for Sensorimotor Network (SMN) Connection Strength Model

Effect	Estimate	SE	t Value	P-Value
**Intercept**	0.1878	0.002940	63.88	<.0001
**AUH**	0.004401	0.002889	1.52	0.1277
**SMN**	0.06974	0.000755	92.41	<.0001
**GE**	0.02639	0.000799	33.04	<.0001
**CC**	0.06715	0.001130	59.43	<.0001
**Degree**	-0.03754	0.000401	-93.55	<.0001
**Modularity**	-0.01136	0.001427	-7.96	<.0001
**GE*AUH**	0.001498	0.001131	1.33	0.1850
**CC*AUH**	-0.00025	0.001599	-0.15	0.8776
**AUH*SMN**	0.001125	0.001044	1.08	0.2812
**GE*SMN**	0.02814	0.001362	20.67	<.0001
**CC*SMN**	0.007240	0.001067	6.79	<.0001
**GE*AUH*SMN**	-0.01663	0.001929	-8.62	<.0001
**CC*AUH*SMN**	0.01991	0.001550	12.85	<.0001
**Dist**	-0.03710	0.000412	-90.11	<.0001
**Dist2**	0.02790	0.000223	125.06	<.0001
**Site A**	0.007202	0.004185	1.72	0.0853
**Site B**	-0.00666	0.004170	-1.60	0.1104
**Site C**	0.000548	0.004338	0.13	0.8994
**Site D**	0.009315	0.004479	2.08	0.0376
**Site E**	0	.	.	.

Table S17: Alcohol use history (AUH): A binary variable representing alcohol use history group (0- no/low, 1-hazardous). Adjusted using the adaptive FDR procedure described in (Benjamini & Hochberg, 2000).

Abbreviations: AUH, alcohol use history; GE, global efficiency; CC, clustering coefficient; Dist, distance; SE, standard error; SMN, sensorimotor network.

Supplemental Table S18- Full mixed-effects results for Salience Network (SN) Connection Probability Model

Effect	Estimate	SE	t Value	P-Value
**Intercept**	-0.2693	0.01383	-19.48	<.0001
**AUH**	0.006032	0.01358	0.44	0.6569
**SN**	0.7704	0.01542	49.97	<.0001
**GE**	0.1694	0.01299	13.04	<.0001
**CC**	-0.2285	0.01565	-14.60	<.0001
**Degree**	-0.08487	0.005403	-15.71	<.0001
**Modularity**	0.03502	0.006699	5.23	<.0001
**GE*AUH**	-0.02777	0.01837	-1.51	0.1306
**CC*AUH**	0.02572	0.02213	1.16	0.2451
**AUH*SN**	0.06687	0.02144	3.12	0.0018
**GE*SN**	0.7098	0.02914	24.36	<.0001
**CC*SN**	-0.2381	0.02656	-8.97	<.0001
**GE*AUH*SN**	-0.00119	0.04130	-0.03	0.9770
**CC*AUH*SN**	0.04005	0.03888	1.03	0.3030
**Dist**	-0.2172	0.003952	-54.96	<.0001
**Dist2**	0.1839	0.002263	81.29	<.0001
**Site A**	0.007069	0.01968	0.36	0.7194
**Site B**	0.009855	0.01960	0.50	0.6152
**Site C**	-0.01264	0.02039	-0.62	0.5354
**Site D**	-0.00586	0.02106	-0.28	0.7810
**Site E**	0	.	.	.

Table S18: Alcohol use history (AUH): A binary variable representing alcohol use history group (0- no/low, 1-hazardous). Adjusted using the adaptive FDR procedure described in (Benjamini & Hochberg, 2000).

Abbreviations: AUH, alcohol use history; GE, global efficiency; CC, clustering coefficient; Dist, distance; SE, standard error; SN, salience network.

Supplemental Table S19- Full mixed-effects results for Salience Network (SN) Connection Strength Model

Effect	Estimate	SE	t Value	P-Value
**Intercept**	0.1888	0.003021	62.49	<.0001
**AUH**	0.004704	0.002969	1.58	0.1131
**SN**	0.04431	0.001484	29.85	<.0001
**GE**	0.02464	0.000728	33.85	<.0001
**CC**	0.07060	0.001000	70.60	<.0001
**Degree**	-0.03803	0.000415	-91.64	<.0001
**Modularity**	-0.01196	0.001466	-8.16	<.0001
**GE*AUH**	0.000239	0.001031	0.23	0.8163
**CC*AUH**	0.001310	0.001416	0.92	0.3550
**AUH*SN**	0.003206	0.002052	1.56	0.1181
**GE*SN**	0.1035	0.002406	43.03	<.0001
**CC*SN**	-0.04155	0.002138	-19.43	<.0001
**GE*AUH*SN**	-0.00159	0.003394	-0.47	0.6385
**CC*AUH*SN**	0.002278	0.003099	0.73	0.4624
**Dist**	-0.03804	0.000417	-91.13	<.0001
**Dist2**	0.02813	0.000224	125.80	<.0001
**Site A**	0.007410	0.004301	1.72	0.0849
**Site B**	-0.00676	0.004285	-1.58	0.1150
**Site C**	0.000920	0.004457	0.21	0.8364
**Site D**	0.01004	0.004603	2.18	0.0292
**Site E**	0	.	.	.

Table S19: Alcohol use history (AUH): A binary variable representing alcohol use history group (0- no/low, 1-hazardous). Adjusted using the adaptive FDR procedure described in (Benjamini & Hochberg, 2000).

Abbreviations: AUH, alcohol use history; GE, global efficiency; CC, clustering coefficient; Dist, distance; SE, standard error; SN, salience network.

Supplemental Table S20- Full mixed-effects results for Visual Network (VN) Connection Probability Model

Effect	Estimate	SE	t Value	P-Value
**Intercept**	-0.2629	0.01409	-18.65	<.0001
**AUH**	0.007542	0.01384	0.54	0.5858
**VN**	1.2654	0.01862	67.97	<.0001
**GE**	0.1901	0.01373	13.84	<.0001
**CC**	-0.2442	0.01666	-14.65	<.0001
**Degree**	-0.08374	0.005419	-15.45	<.0001
**Modularity**	0.03576	0.006828	5.24	<.0001
**GE*AUH**	-0.02992	0.01942	-1.54	0.1234
**CC*AUH**	0.02984	0.02357	1.27	0.2055
**AUH*VN**	-0.1095	0.02560	-4.28	<.0001
**GE*VN**	-0.09679	0.03739	-2.59	0.0096
**CC*VN**	0.5976	0.03350	17.84	<.0001
**GE*AUH*VN**	0.1504	0.05072	2.97	0.0030
**CC*AUH*VN**	-0.2611	0.04617	-5.65	<.0001
**Dist**	-0.2110	0.003918	-53.87	<.0001
**Dist2**	0.1789	0.002270	78.78	<.0001
**Site A**	0.007287	0.02006	0.36	0.7164
**Site B**	0.008636	0.01998	0.43	0.6656
**Site C**	-0.01312	0.02078	-0.63	0.5279
**Site D**	-0.00554	0.02147	-0.26	0.7962
**Site E**	0	.	.	.

Table S20: Alcohol use history (AUH): A binary variable representing alcohol use history group (0- no/low, 1- hazardous). Adjusted using the adaptive FDR procedure described in (Benjamini & Hochberg, 2000).

Abbreviations: AUH, alcohol use history; GE, global efficiency; CC, clustering coefficient; Dist, distance; SE, standard error; VN, visual network.

Supplemental Table S21- Full mixed-effects results for Visual Network (VN) Connection Strength Model

Effect	Estimate	SE	t Value	P-Value
**Intercept**	0.1891	0.003033	62.34	<.0001
**AUH**	0.004898	0.002981	1.64	0.1003
**VN**	0.1997	0.001258	158.82	<.0001
**GE**	0.02995	0.000679	44.13	<.0001
**CC**	0.06548	0.000968	67.67	<.0001
**Degree**	-0.03746	0.000421	-89.06	<.0001
**Modularity**	-0.01159	0.001472	-7.87	<.0001
**GE*AUH**	-0.00104	0.000961	-1.08	0.2784
**CC*AUH**	0.002881	0.001370	2.10	0.0355
**AUH*VN**	-0.00342	0.001757	-1.94	0.0519
**GE*VN**	0.01731	0.002503	6.91	<.0001
**CC*VN**	0.06129	0.002029	30.21	<.0001
**GE*AUH*VN**	0.03491	0.003501	9.97	<.0001
**CC*AUH*VN**	-0.00537	0.002965	-1.81	0.0700
**Dist**	-0.03575	0.000409	-87.41	<.0001
**Dist2**	0.02661	0.000220	120.73	<.0001
**Site A**	0.007339	0.004318	1.70	0.0892
**Site B**	-0.00686	0.004303	-1.59	0.1107
**Site C**	0.001008	0.004475	0.23	0.8218
**Site D**	0.009904	0.004622	2.14	0.0321
**Site E**	0	.	.	.

Table S21: Alcohol use history (AUH): A binary variable representing alcohol use history group (0- no/low, 1- hazardous). Adjusted using the adaptive FDR procedure described in (Benjamini & Hochberg, 2000).

Abbreviations: AUH, alcohol use history; GE, global efficiency; CC, clustering coefficient; Dist, distance; SE, standard error; VN, visual network.

Supplemental References

Amaratunga, D., & Cabrera, J. (2001). Analysis of Data from Viral DNA Microchips. *Journal of the American Statistical Association*, *96*, 1161-1170. https://doi.org/10.1198/016214501753381814

Bahrami, M., Laurienti, P. J., & Simpson, S. L. (2019a). Analysis of brain subnetworks within the context of their whole-brain networks. *Human Brain Mapping*, *40*(17), 5123-5141. <Go to ISI>://WOS:000483163900001

Bahrami, M., Laurienti, P. J., & Simpson, S. L. (2019b). A MATLAB toolbox for multivariate analysis of brain networks. *Human Brain Mapping*, *40*(1), 175-186. https://doi.org/10.1002/hbm.24363

Bahrami, M., Simpson, S. L., Burdette, J. H., Lyday, R. G., Quandt, S. A., Chen, H., Arcury, T. A., & Laurienti, P. J. (2022). Altered default mode network associated with pesticide exposure in Latinx children from rural farmworker families. *NeuroImage*, *256*, 119179. https://doi.org/10.1016/j.neuroimage.2022.119179

Benjamini, Y., & Hochberg, Y. (2000). On the Adaptive Control of the False Discovery Rate in Multiple Testing With Independent Statistics. *Journal of Educational and Behavioral Statistics*, *25*(1), 60-83. https://doi.org/10.3102/10769986025001060

Bolstad, B. M., Irizarry, R. A., Astrand, M., & Speed, T. P. (2003). A comparison of normalization methods for high density oligonucleotide array data based on variance and bias. *Bioinformatics*, *19*(2), 185-193. https://doi.org/10.1093/bioinformatics/19.2.185

Fraiman, D., Balenzuela, P., Foss, J., & Chialvo, D. R. (2009). Ising-like dynamics in large-scale functional brain networks. *Phys Rev E Stat Nonlin Soft Matter Phys*, *79*(6 Pt 1), 061922. https://doi.org/10.1103/PhysRevE.79.061922

Laurienti, P. J., Miller, M. E., Lyday, R. G., Boyd, M. C., Tanase, A. D., Burdette, J. H., Hugenschmidt, C. E., Rejeski, W. J., Simpson, S. L., Baker, L. D., Tomlinson, C. E., & Kritchevsky, S. B. (2023). Associations of physical function and body mass index with functional brain networks in community-dwelling older adults. *Neurobiol Aging*, *127*, 43-53. https://doi.org/10.1016/j.neurobiolaging.2023.03.008

Parente, F., Frascarelli, M., Mirigliani, A., Di Fabio, F., Biondi, M., & Colosimo, A. (2018). Negative functional brain networks. *Brain Imaging Behav*, *12*(2), 467-476. https://doi.org/10.1007/s11682-017-9715-x

Pruim, R. H. R., Mennes, M., van Rooij, D., Llera, A., Buitelaar, J. K., & Beckmann, C. F. (2015). ICA-AROMA: A robust ICA-based strategy for removing motion artifacts from fMRI data. *NeuroImage*, *112*, 267-277. https://doi.org/10.1016/j.neuroimage.2015.02.064

Shen, X., Tokoglu, F., Papademetris, X., & Constable, R. T. (2013). Groupwise whole-brain parcellation from resting-state fMRI data for network node identification. *NeuroImage*, *82*, 403-415. https://doi.org/10.1016/j.neuroimage.2013.05.081

Simpson, S. L., & Laurienti, P. J. (2015). A two-part mixed-effects modeling framework for analyzing whole-brain network data. *Neuroimage*, *113*, 310-319. https://doi.org/10.1016/j.neuroimage.2015.03.021
